# A non-canonical mismatch repair pathway in prokaryotes

**DOI:** 10.1038/ncomms14246

**Published:** 2017-01-27

**Authors:** A. Castañeda-García, A. I. Prieto, J. Rodríguez-Beltrán, N. Alonso, D. Cantillon, C. Costas, L. Pérez-Lago, E. D. Zegeye, M. Herranz, P. Plociński, T. Tonjum, D. García de Viedma, M. Paget, S. J. Waddell, A. M. Rojas, A. J. Doherty, J. Blázquez

**Affiliations:** 1 Stress and Bacterial Evolution Group, Instituto de Biomedicina de Sevilla. Avda. Manuel Siurot S/N, 41013-Sevilla, Spain; 2 Genome Damage and Stability Centre, School of Life Sciences, University of Sussex, Brighton BN1 9RQ, UK; 3 Centro Nacional de Biotecnología-CSIC. C/ Darwin 3, 28049-Madrid, Spain; 4 Brighton and Sussex Medical School, University of Sussex, Brighton BN1 9PX, UK; 5 Servicio de Microbiología Clínica y Enfermedades Infecciosas, Hospital Gregorio Marañón and Instituto de Investigación Sanitaria Gregorio Marañón. Dr. Esquerdo 46, 28007-Madrid, Spain; 6 Department of Microbiology, Oslo University Hospital, Rikshospitalet, Oslo, Norway and Department of Microbiology, University of Oslo, P.O. Box 1072 Blindern, 0316 Oslo, Norway; 7 School of Life Sciences, University of Sussex, Brighton BN1 9QG, UK; 8 Computational Biology and Bioinformatics, Instituto de Biomedicina de Sevilla (IBIS)-CSIC. Avda. Manuel Siurot S/N, 41013-Sevilla Spain; 9 Unit of Infectious Diseases, Microbiology, and Preventive Medicine. University Hospital Virgen del Rocio, Avda. Manuel Siurot S/N, 41013-Sevilla, Spain

## Abstract

Mismatch repair (MMR) is a near ubiquitous pathway, essential for the maintenance of genome stability. Members of the MutS and MutL protein families perform key steps in mismatch correction. Despite the major importance of this repair pathway, MutS–MutL are absent in almost all Actinobacteria and many Archaea. However, these organisms exhibit rates and spectra of spontaneous mutations similar to MMR-bearing species, suggesting the existence of an alternative to the canonical MutS–MutL-based MMR. Here we report that *Mycobacterium smegmatis* NucS/EndoMS, a putative endonuclease with no structural homology to known MMR factors, is required for mutation avoidance and anti-recombination, hallmarks of the canonical MMR. Furthermore, phenotypic analysis of naturally occurring polymorphic NucS in a *M. smegmatis* surrogate model, suggests the existence of *M. tuberculosis* mutator strains. The phylogenetic analysis of NucS indicates a complex evolutionary process leading to a disperse distribution pattern in prokaryotes. Together, these findings indicate that distinct pathways for MMR have evolved at least twice in nature.

## Abstract (web summary)

Despite the importance of mismatch repair for genome stability, many Archaea and almost all Actinobacteria lack MutS and MutL proteins. Here the authors, using *Mycobacterium smegmatis* as a model, report that NucS/EndoMS endonuclease acts in a distinct mismatch repair pathway.

Cells ensure the maintenance of genome stability and a low mutation rate using a plethora of DNA surveillance and correction processes. These include base selection, proofreading, mismatch repair (MMR), base/nucleotide excision repair, recombination repair and non-homologous end-joining^1^. The recognized MMR pathway is highly conserved among the three domains of life^2^. In all cases, members of the MutS and MutL protein families perform key steps in mismatch correction. This MutS–MutL-based MMR system (canonical MMR) is a sophisticated DNA repair pathway that detects and removes incorrect mismatched nucleobases. Mismatched base pairs in DNA arise mainly as a result of DNA replication errors due to the incorporation of wrong nucleobases by DNA polymerases. To correct them, the system detects incorrect although chemically normal bases that are mismatched with the complementary strand, discriminating between the parental template and the newly synthesized strand^3^.

Loss of this activity has very important consequences, such as high rates of mutation (hypermutability) and increased recombination between non perfectly identical (homeologous) DNA sequences^4^, both hallmarks of MMR inactivation. Nevertheless, despite the *quasi*-ubiquitous nature of this pathway, there are some important exceptions. The genomes of many Archaea, including Crenarchaeota and a few groups of Euryarchaeota, and almost all members of the bacterial phylum Actinobacteria, including *Mycobacterium*, have been shown to possess no identifiable MutS or MutL homologues^5^,^6^,^7^. However, these prokaryotes exhibit rates and spectra of spontaneous mutations similar to canonical MMR-bearing bacterial species^8^,^9^,^10^, suggesting the existence of unidentified mechanisms responsible for mismatch repair.

To identify novel mutation avoidance genes in mycobacteria, we performed a genetic screen and discovered that inactivation of the *MSMEG_4923* gene in *Mycobacterium smegmatis*, encoding a homologue of an archaeal endonuclease dubbed NucS (for nuclease specific for ssDNA)^11^, produced a hypermutable phenotype. NucS from *Pyrococcus abyssi* was initially identified as a member of a new family of novel structure-specific DNA endonucleases in Archaea^11^. Notably, a recent report revealed that the protein NucS from the archaeal species *Thermococcus kodakarensis* (renamed as EndoMS) is a mismatch-specific endonuclease, acting specifically on double-stranded DNA (dsDNA) substrates containing mismatched bases^12^. The possibility that a non-canonical mismatch repair could be triggered by a specific double-strand break (DSB) endonuclease, able to act at the site of a mispair, followed by DSB repair was hypothesized 30 years ago^13^. The archaeal NucS binds and cleaves both strands at dsDNA mismatched substrates, with the mispaired bases in the central position, leaving 5-nucleotide long 5′-cohesive ends^12^. Consequently, it has been suggested that these specific DSBs may promote the repair of mismatches, acting in a novel MMR process^12^. Moreover, very recently, the structure of the *T. kodakarensis* NucS-mismatched dsDNA complex has been determined, strongly supporting the idea that NucS acts in a mismatch repair pathway^14^. Although these biochemical and structural studies have shed some light on the role of NucS at the molecular level, the cellular and biological function of NucS and its impact on genome stability remains unknown.

Hypermutable bacterial pathogens, very often associated with defects in MMR components, are frequently isolated and pose a serious risk in many clinical infections^15^,^16^,^17^,^18^,^19^,^20^,^21^. The major pathogen *Mycobacterium tuberculosis* appears to be genetically isolated, acquires antibiotic resistance exclusively through chromosomal mutations^22^ and presents variability in mutation rates between strains^23^. These factors should contribute to the selection of hypermutable *M. tuberculosis* strains under antibiotic pressure, as it occurs with other chronic pathogens^18^,^23^. However, hypermutable strains have not yet been detected in this pathogen. To investigate the possible existence of *M. tuberculosis* hypermutable strains affected in NucS activity, we analysed the effect of naturally occurring polymorphisms in *nucS* from *M. tuberculosis* clinical isolates on mutation rates, using *M. smegmatis* as a surrogate model.

In this study, we first establish that NucS is essential for maintaining DNA stability, by preventing the acquisition of DNA mutations in *Mycobacterium* and *Streptomyces*. Inactivation of *nucS* in *M. smegmatis* produces specific phenotypes that mimic those of canonical MMR-null mutants (increased mutation rate, biased mutational spectrum and increased homeologous recombination). Finally, we conduct here distribution and phylogenetic analyses of NucS across the prokaryotic domains to understand its evolutionary origins.

## Results

### Identification and characterization of *M. smegmatis nucS*

**Figure 1:**
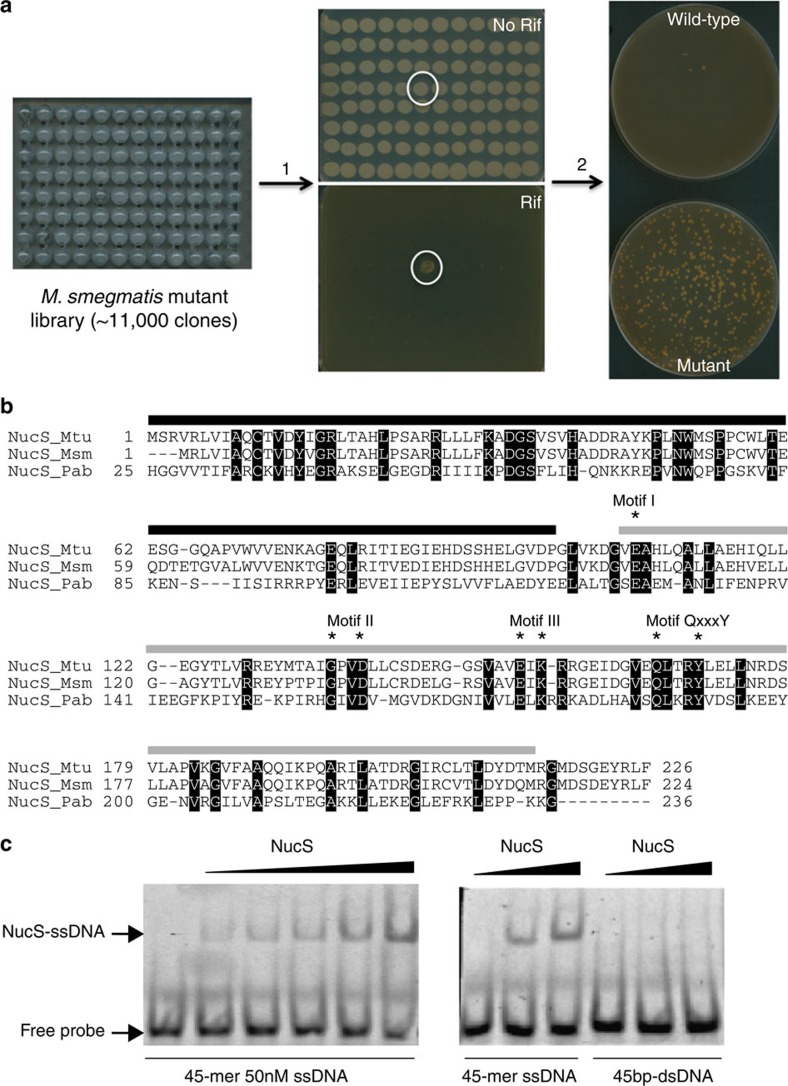
Identification and characterization of *M. smegmatis* NucS. (**a**) Schematic representation of the process for identifying the *nucS* transposon mutant. ∼11,000 clones from the *M. smegmatis* insertion mutant library were replicated onto plates with (Rif) or without (No Rif) rifampicin (step 1). One single clone (circled) produced a high number of Rif-R colonies. After isolation and purification (step 2), the frequency of spontaneous Rif-R mutants (bottom plate) was checked and compared with that of the wild-type (upper plate), demonstrating its hypermutable phenotype. (**b**) Multiple sequence alignment of NucS sequences. *M. tuberculosis* (NucS_Mtu), *M. smegmatis* (NucS_Msm) and *P. abyssi* (NucS_Pab) sequences are from Uniprot (identifiers are P9WIY4, A0R1Z0 and Q9V2E8, respectively). Solid lines over the alignment indicate protein domains as defined previously for *P. abyssi* NucS^11^ (black, DNA-binding; grey, nuclease). Identical amino acid residues are shown in black. Catalytic residues required for nuclease activity in *P. abyssi* NucS^11^ are labelled with asterisks. Nuclease motifs of *P. abyssi* NucS are also indicated^11^. (**c**) DNA-binding activity of NucS. In a gel-based EMSA, purified NucS protein (1–16 μM) is capable of binding to 45-mer ssDNA (50 nM) (left side) but not to 45-bp dsDNA (50 nM) (right side). The arrow indicates the position of the DNA–NucS complex.

To identify novel mutation avoidance genes in mycobacteria, a *M. smegmatis* mc^2^ 155 library of ∼11,000 independent transposon insertion mutants was generated and screened for spontaneous mutations that confer rifampicin resistance (Rif-R), used as a hypermutator hallmark (Fig. 1a). One transposon insertion, which inactivated the *MSMEG_4923* (*nucS*) gene, conferred a strong hypermutable phenotype. The NucS/EndoMS translated protein sequence is 27% identical to NucS from the hyperthermophilic archaeal *P. abyssi* and 87% to that of *M. tuberculosis* (Fig. 1b). *P. abyssi* NucS was defined as the first member of a new family of structure-specific endonucleases with a mismatch-specific endonuclease activity^11^,^12^ containing an N-terminal DNA-binding domain and a C-terminal RecB-like nuclease domain^11^. All the catalytic residues required for nuclease activity in *P. abyssi* are conserved in the mycobacterial NucS (Fig. 1b).

Recombinant *M. smegmatis* NucS was expressed and purified. The apparent molecular mass of the purified native protein fits with the expected size (25 kDa) (Supplementary Fig. 1A). The biochemical activity was analysed by DNA electrophoretic mobility shift assays (EMSAs) and nuclease enzymatic assays. *M. smegmatis* NucS was capable of binding to single-stranded DNA (ssDNA), as seen in the archaeal *P. abyssi* NucS^11^, but not to dsDNA (Fig. 1c). Regarding cleavage of mismatched substrates, no significant specific cleavage activity was observed on different substrates containing a single nucleotide mismatch in the central region of the strand (Supplementary Fig. 1B), in contrast with previous results obtained with the archaeal *T. kodakarensis* NucS^12^. The binding to ssDNA indicates that the protein is correctly folded, as it can bind to its substrate. This suggests that different requirements, such as the binding of additional partners, protein modifications or different DNA substrates, may be required to activate the mismatch-specific cleavage activity of the bacterial NucS.

### NucS is essential to maintain low levels of spontaneous mutation

**Figure 2:**
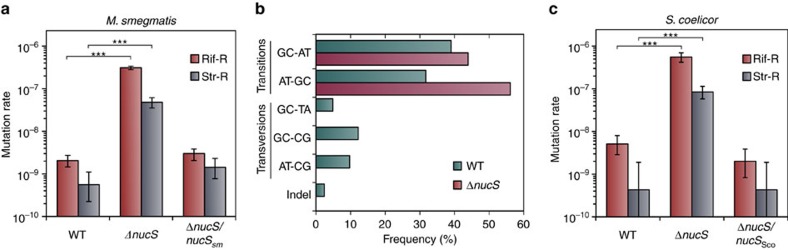
Mutational effects of *nucS* deletion. (**a**) Rates of spontaneous mutations conferring rifampicin, Rif-R (red), and streptomycin resistance, Str-R (grey) of *M. smegmatis* mc^2^ 155 (WT), its Δ*nucS* derivative and the Δ*nucS* strain complemented with *nucS* from *M. smegmatis* mc^2^ 155 (*nucS*_Sm_). (**b**) Mutational spectrum of *M. smegmatis* mc^2^ 155 (green) and its Δ*nucS* derivative (red). Bars represent the frequency of the types of change found in *rpoB*. (**c**) Rates of spontaneous mutations conferring Rif-R (red) and Str-R (grey) of *S. coelicolor* A3(2) M145 (WT), its *ΔnucS* derivative and the Δ*nucS* strain complemented with the wild-type *nucS* from *S. coelicolor* (*nucS*_Sco_). Error bars represent 95% confidence intervals (*n*=20). Asterisks denote statistical significance (Likelihood ratio test under Luria-Delbruck model, Bonferroni corrected, *P* value <10^−4^ in all cases). Mutation rate: mutations per cell per generation.

To verify that NucS has a key role in maintaining DNA fidelity, we constructed an in-frame deletion of *nucS* (Δ*nucS, nucS-*null derivative) in *M. smegmatis* and measured the rate at which drug resistance was acquired. Notably, the *nucS*-null strain displayed a hypermutable phenotype, increasing the rate of spontaneously emerging rifampicin and streptomycin (Str-R) resistances by a factor of 150-fold and 86-fold, respectively, above the wild-type strain (3.1 × 10^−7^ versus 2.1 × 10^−9^ and 4.8 × 10^−8^ versus 5.6 × 10^−10^, Δ*nucS* versus wild type, respectively). These results are equivalent to those observed for *mutS*- or *mutL-*deficient *Escherichia coli* (10^2^–10^3^-fold increases)^24^. Significantly, basal mutation rates were recovered when the *nucS* deletion was chromosomally complemented with the wild-type gene *MSMEG_4923* (*nucS*_Sm_) (Fig. 2a, Supplementary Table 1), confirming that inactivation of this gene was responsible for the high mutation rates observed.

Notably, *M. smegmatis* mc^2^ 155 and its Δ*nucS* derivative produced a different mutational signature. While spontaneous Rif-R mutations detected in the wild-type strain comprise different base substitutions, including transitions, but also transversions and even an in-frame deletion, all mutations detected in the Δ*nucS* strain were specifically transitions (A:T→G:C or G:C→A:T) (Fig. 2b, Supplementary Tables 2A,B). Transitions occur much more frequently than transversions or indels in any cell, due to spontaneous or induced errors in the DNA, and are the preferred substrates for MMR mechanism. The Δ*nucS* strain accumulates a high number of uncorrected transitions, masking transversions and indels to undetectable levels under our experimental conditions. This transition-biased mutational spectrum is a hallmark signature of canonical MMR pathway^25^,^26^.

To demonstrate the generality of the mutation-avoidance activity in species encoding NucS, the *nucS* gene was deleted in *Streptomyces coelicolor* A3(2), a different actinobacterial species. The precise deletion of *nucS* in this species increased the rate of spontaneous mutations conferring Rif-R and Str-R by a factor of 108-fold and 197-fold, respectively. As with *M. smegmatis*, low mutation rates were recovered by complementation with the wild-type *S. coelicolor nucS* gene (Fig. 2c, Supplementary Table 3). Together, these results highlight the essential role of NucS in mutation avoidance.

### NucS inhibits homeologous but not homologous recombination

**Figure 3:**
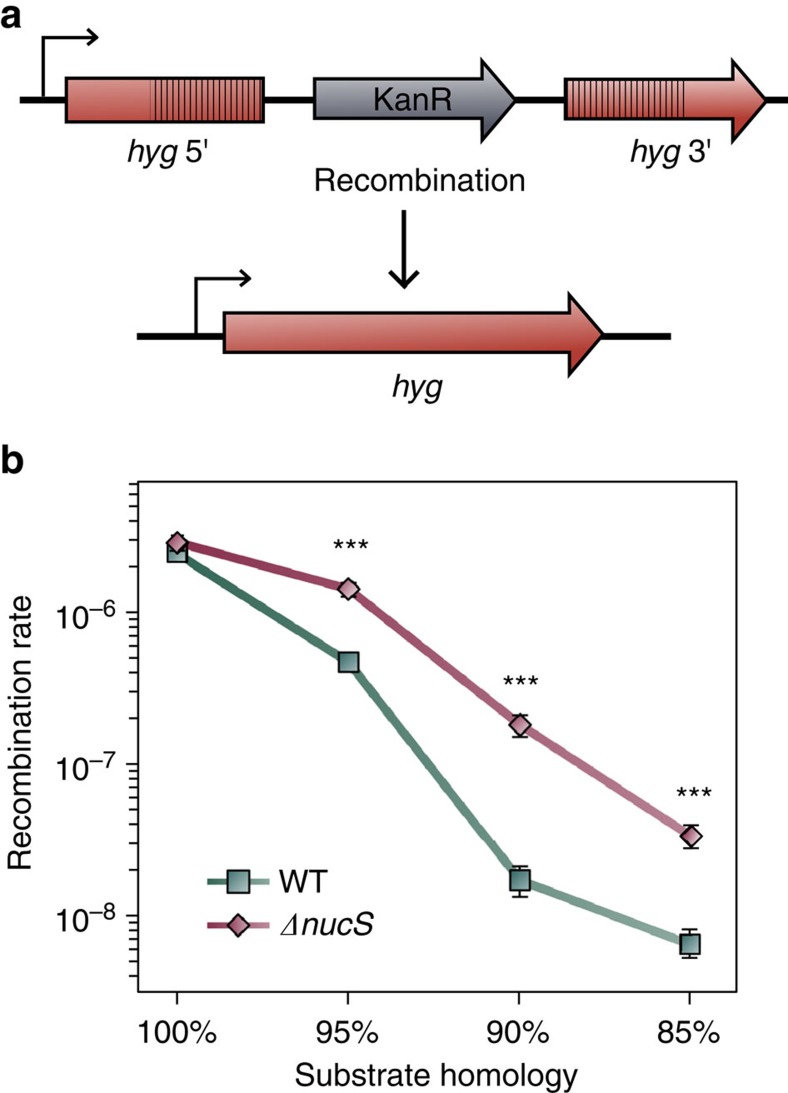
Effect of *nucS* deletion on recombination. (**a**) Chromosomal construct used to measure recombination between homologous or homeologous DNA sequences. The *hyg* gene is reconstituted by a single recombination event between two 517-bp overlapping fragments (striped), sharing different degree of sequence identity (100%, 95%, 90% and 85%) and separated by a kanamycin resistant (Kan-R) gene. Recombinant clones express hygromycin resistance and kanamycin susceptibility. (**b**) Rates of recombination between homologous and homeologous DNA sequences with different degree of identity (%) in *M. smegmatis* mc^2^ 155 (WT, green squares) and its Δ*nucS* derivative (red diamonds). Error bars represent 95% confidence intervals (*n*=16). Asterisks denote statistical significance (Likelihood ratio test under Luria–Delbruck model, Bonferroni corrected, *P* value <10^−4^ in all cases).

These results prompted us to investigate whether NucS is involved in reduction of recombination between non-identical (homeologous) DNA sequences, but not between 100% identical, as described for the canonical MMR-null mutants in other bacterial species^4^,^27^. The rates of recombination between homologous and homeologous sequences were measured using specific engineered tools (Fig. 3a, Supplementary Fig. 2A,B). Recombination rates between 100% identical sequences were similar in the wild-type and its Δ*nucS* derivative (2.42 × 10^−6^ and 2.86 × 10^−6^, respectively). However, when the identity of DNA sequences decreased, the recombination rate was comparatively higher in the *nucS*-null derivative: 95%, 4.68 × 10^−7^ versus 1.41 × 10^−6^ (3-fold difference), 90%, 1.71 × 10^−8^ versus 1.82 × 10^−7^ (10-fold) and 85%, 6.47 × 10^−9^ versus 3.36 × 10^−8^ (5-fold) for wild type versus Δ*nucS*, respectively (Fig. 3b). We verified that recombinants detected were exclusively due to recombination events but not spontaneous mutation (see ‘Methods' section). The inhibitory effect of NucS on homeologous but not homologous recombination again resembles an important tell-tale signature of a canonical MMR pathway^28^,^29^.

### NucS polymorphisms in *M. tuberculosis* clinical strains

**Figure 4:**
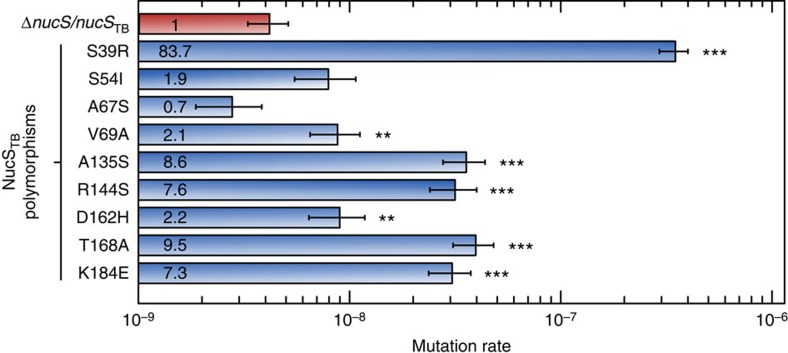
Effects of NucS polymorphisms on mutation rates in the *M. smegmatis* Δ*nucS* surrogate model. Rates of spontaneous mutations conferring Rif-R of the *M. smegmatis* Δ*nucS* complemented with wild-type *nucS*_TB_ (Δ*nucS*/*nucS*_TB_; red) or containing each of the nine polymorphisms indicated (blue). Relative increases in mutation rates with respect to the control strain (Δ*nucS*/*nucS*_TB_; set to 1) are shown inside the column. Error bars represent 95% confidence intervals (*n*=20). Asterisks denote statistical significance (Likelihood ratio test under Luria-Delbruck model, Bonferroni corrected; ***P<0.001; **P<0.005). Mutation rate: mutations per cell per generation.

Once the possibility of hypermutability was demonstrated in *M. smegmatis*, we searched for the existence of *M. tuberculosis* hypermutable clinical isolates by *nucS* inactivation. Analysis of the *nucS*_TB_ sequences from ∼1,600 clinical *M. tuberculosis* strains available in the databases revealed a total of nine missense single nucleotide polymorphisms (SNPs) (Supplementary Table 4, Supplementary Fig. 3). The effects of these polymorphisms on NucS activity were experimentally analysed by checking their impact on mutation rates, using a *M. smegmatis* heterologous system, as previously reported for other mutagenesis studies^30^. Ten *nucS*_TB_ alleles (nine polymorphic plus the wild type) were integrated into the chromosome of *M. smegmatis* Δ*nucS*. Complementation with wild-type *nucS*_TB_ restored low mutation rates to *M. smegmatis* Δ*nucS*. However, five alleles increased mutation rate significantly (Fig. 4, Supplementary Table 5), with allele S39R presenting the strongest observed mutator phenotype (83-fold increase). Alleles A135S, R144S, T168A and K184E produced increases in mutation rates close to one order of magnitude, whereas alleles S54I, A67S, V69A and D162H produced low increases (∼2) or no changes. These results suggest the existence of hypermutable *M. tuberculosis* clinical strains affected by modulation of NucS activity.

### NucS taxonomic distribution

Taken together, our results compellingly suggest a functional connection between NucS and known MMR pathways. We analysed the species distribution of NucS taking also into account the canonical MMR proteins, MutS and MutL (for MutS and MutL analyses see Supplementary Methods). A total of 3,942 reference proteomes were scanned for presence/absence of NucS (Supplementary Data 1, Supplementary Fig. 4). Bacteria are represented by 2,709 proteomes (68%) and Archaea by 132 (4%), the remaining 1,101 (28%) belonging to Eukaryota and Virus. NucS is present in 370 organisms, from the domains Archaea (60 species) and Bacteria (310 species) (Supplementary Data 1). It is totally absent from eukaryotes and virus.

**Figure 5:**
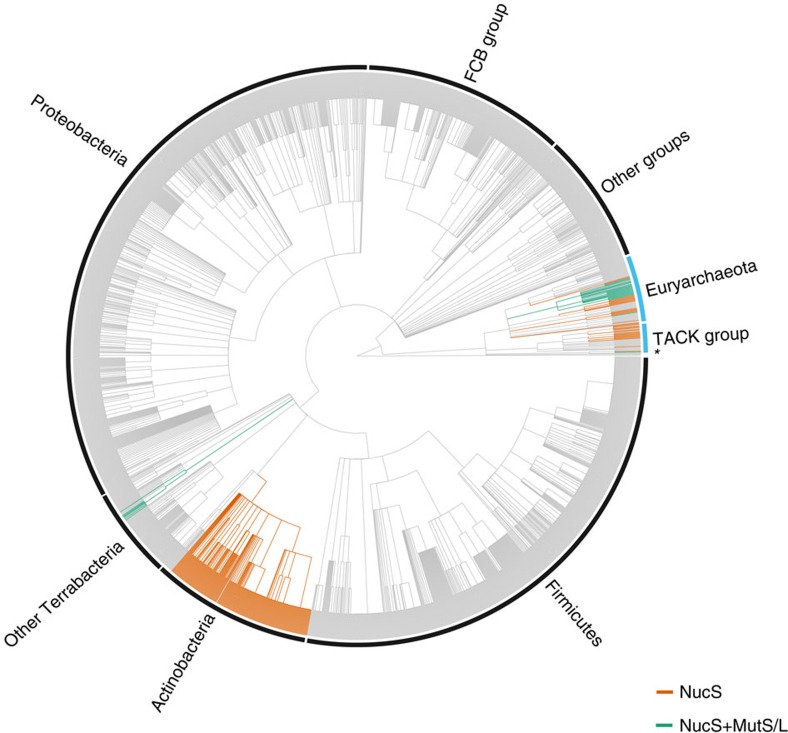
Phylogenetic profiling of NucS. The NCBI taxonomic tree from 2,186 species from Bacteria (black outer label) and Archaea (blue outer label). Orange branches: NucS only; green branches: NucS and MutS–MutL. Bacteria includes Actinobacteria, Firmicutes, Proteobacteria, FCB (Fibrobacteres, Chlorobi and Bacteroidetes), Other Terrabacteria (Armatimonadetes, Chloroflexi, Cyanobacteria, Deinococcus-Thermus, Tenericutes and unclassified Terrabacteria) and other groups (Acidobacteria, Aquificae, Caldiserica, Chrysiogenetes, Deferribacteres, Dictyoglomi, Elusimicrobia, Fusobacteria, Nitrospirae, PVC group, Spirochaetes, Synergistetes, Thermodesulfobacteria, Thermotogae and unclassified bacteria). Archaea includes Euryarchaeota, TACK (Thaumarchaeota, Aigarchaeota, Crenarchaeota and Korarchaeota) and unclassified archaeal species (*). As NucS is absent in eukaryotes and viruses, these lineages were removed for clarity purposes. The tree was annotated using ggtree (http://www.bioconductor.org/packages/ggtree).

To highlight the distribution of NucS in all organisms, we built a phylogenetic profile of NucS. Figure 5 shows this profile mapped onto the NCBI taxonomy tree, containing 2,186 bacterial and archaeal species (Supplementary Data 2). The NucS distribution pattern indicates that this protein exhibits a disperse distribution (Fig. 5, Supplementary Data 1).

In Bacteria, the phylum Actinobacteria is the one containing the majority of NucS with 300 species in the class Actinobacteria (only two exceptions) and three species in other classes of the phylum (Supplementary Data 1), *Conexibacter woesei* (class Thermoleophilia), *Patulibacter medicamentivorans* (class Thermoleophilia) and *Ilumatobacter coccineus* (class Acidimicrobiia). All the analysed members of the class Coriobacteriia, from the Actinobacteria phylum, lack NucS and MutS–MutL proteins, while the two species from the class Rubrobacteria lack NucS, but present MutS–MutL. The pattern is even more disperse in Archaea. From 132 species, 60 have NucS (21 also contain MutS–MutL). NucS, but not MutS–MutL, is present in 17 out of 24 species of the phylum Crenarchaeota and in 18 out of 88 of the phylum Euryarchaeota. By contrast, MutS–MutL (but not NucS) is completely absent in crenarchaeotal species while it is restricted to 29 euryarchaeotal species (Supplementary Data 1).

Interestingly, only 28 organisms, 21 halobacterial species (domain Archaea) and 7 species of the phylum Deinococcus-Thermus (domain Bacteria), have both NucS and MutS–MutL sequences. Notably, when we focused our analysis in the two main NucS-containing groups (Actinobacteria and Archaea), some species still lack both NucS and MutS–MutL (confirmed by protein translated searches -tblastn- in Actinobacteria and Archaea, Supplementary Data 1).

### A model for the origin and evolution of NucS

Our results support a complex evolutionary ancestry for NucS. Comprehensive protein sequence analyses of NucS indicate that the protein contains two distinct regions, a DNA-binding N-terminal domain (NucS-NT) and an endonuclease C-terminal domain (NucS-CT) (Supplementary Figs 3 and 4), in agreement with previous structural studies^11^. At the sequence level, using profile hidden Markov models (HMMs), we also detected these regions in alternative proteins outside the context of NucS, supporting the original independence of these domains, which have been subsequently fused during evolution.

To understand the particular distribution observed in the phylogenetic profile, we conducted sequence and phylogenetic independent analyses of full NucS and also the N-terminal and C-terminal regions (Supplementary Figs 5–7). The actinobacterial NucS representatives are well separated from those of Archaea. On the other hand, the NucS from Deinococcus-Thermus species group together with the archaeal proteins, instead of the actinobacterial orthologues, suggesting that NucS has recently been transferred from Archaea to these Deinococcus-Thermus species (Supplementary Figs 5–7).

The sequence analyses provide different distributions for the two regions of NucS. While the N-terminal region is limited exclusively to Archaea and some Bacteria (Supplementary Fig. 7), the C-terminal region was found in many archaeal species, some Bacteria, and in a few eukaryotes (Supplementary Fig. 6) and, importantly, also in different domain architectures. These observations, in the context of our phylogenetic analyses, suggest that both regions may have emerged in Archaea. Subsequently, the C-terminal region may have been transferred to some bacterial species and to a few eukaryotes, and got fixed in different protein contexts. The full protein and the individual domains likely got lost in certain groups during the evolution of Archaea. However, the reconstruction of the precise evolution of these individual domains requires deeper analyses in the context of related protein domains.

**Figure 6:**
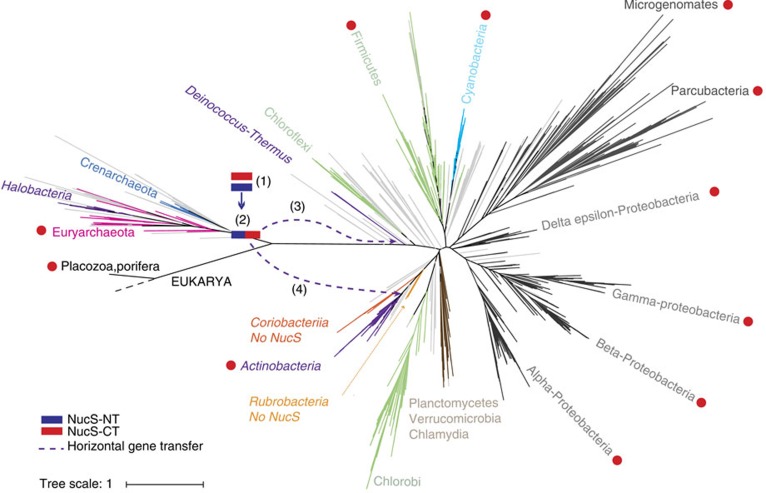
A model for NucS protein emergence and evolution. The unrooted Tree of Life (available and based on ref. 50) was used to depict the proposed evolutionary history of NucS according to our data. The groups relevant to our model are highlighted. Coloured squares depict the NucS-NT (blue) and NucS-CT (red) terminal regions. This model proposes that NucS has an archaeal origin and emerged as a combination of two independent protein domains with complex evolutionary history. Numbers indicate the steps of the model: Both N-terminal and C-terminal regions likely emerged in the archaeal lineage (1). The CT region was transferred via HGT to very few Eukaryotes and to some Bacteria (main groups with any species having the NucS-CT region are labelled with red circles), where the CT domain combined with other regions outside the context of NucS. In the archaeal lineage, NT and CT regions fused to produce the full NucS (2). NucS expanded in many archaeal groups but was also lost in some others. The full NucS protein was transferred to Bacteria by at least two independent HGT events, one to some Deinoccocus-Thermus species (3) and another to Actinobacteria (4).

Phylogenetic analysis of the full protein suggests that NucS emerged after the archaeal divergence by a rearrangement of these domains and then it was transferred from Archaea to certain Deinococcus-Thermus species in at least one event. For Actinobacteria, the most parsimonious explanation suggests a horizontal transfer event of NucS from Archaea coupled with a likely MutS–MutL loss event in the last common ancestor of Actinobacteria (as most of the contemporary NucS-encoding species lack MutS and MutL). This is consistent with the fact that we did not find any species from the entire Bacteria group, except those from Actinobacteria and the Deinococcus-Thermus group, having NucS or NucS-like proteins. Our model (Fig. 6) favours the simplest scenario to explain the complex pattern distribution of the full NucS protein and its absence in all eukaryotes, the vast majority of bacteria, and some archaeal organisms without invoking unlikely massive losses. This is in agreement with our phylogenetic observations.

## Discussion

This study provides genetic and biological evidence that establish the existence of a DNA repair system that mimics the canonical MutS–MutL-based MMR pathway.

To date, the correction of mismatched nucleobases has been defined as a highly conserved mechanism whose key steps are performed, in all cases, by members of the MutS and MutL protein families. Despite the genomes of Crenarchaeota, a few groups of Euryarchaeota and almost all members of the phylum Actinobacteria lacking identifiable MutS or MutL homologues^5^,^6^,^7^, they exhibit rates and spectra of spontaneous mutations similar to canonical MMR-bearing bacterial species^8^,^9^,^10^, suggesting the existence of still undetected pathway responsible for this type of correction. Although recent biochemical and structural reports suggested the existence of a novel mismatch-specific endonuclease, NucS, in the archaeal species *T. kodakarensis*^12^,^14^ that is able to recognize and cleave mismatched bases in dsDNA *in vitro*, no genetic and/or biological evidence had been reported to date on the activity of a novel mismatch repair pathway.

The screening of a large library of *M. smegmatis* mutants revealed that NucS is a key mutation avoidance component in Actinobacteria. Through genetic and biological analysis, we demonstrate that the *nucS*-null phenotypes in *M. smegmatis* are almost identical to those produced by the MMR deficiency in other bacteria (very high mutation rates, transition-biased mutational spectrum and increased homeologous recombination rates). The anti-mutator nature of NucS is also demonstrated in *Streptomyces coelicolor*, a different species of the class Actinobacteria. Therefore, NucS appears to be an important DNA repair factor which, together with the high-fidelity DNA-polymerase DnaE1 and its PHP domain-proofreader^30^, maintain low mutation rates (∼10^−10^ mutations per base per generation^9^,^10^), ensuring genome stability and DNA fidelity in mycobacteria and in other Actinobacteria.

Although SNPs in DNA repair/replication genes have been suggested as a source of hypermutation in *M. tuberculosis*^31^,^32^, only small increases in mutation rates have been observed due to these polymorphic genes (for example, polymorphisms in PHP exonuclease domain of the DNA-polymerase DnaE1 (ref. 30)). Our results suggest the existence of naturally occurring hypermutable *M. tuberculosis* variants with diminished NucS activity. Whether or not this is relevant for the virulence and adaptation to antibiotic treatments remains to be deciphered.

Although we observed that purified mycobacterial NucS binds to ssDNA but not to dsDNA, as described for archaeal NucS proteins^11^,^12^, no significant specific cleavage activity was observed on mismatched substrates, suggesting that activation by other partners and/or modifications (for example, post-translational modifications) is required in *M. smegmatis*. Also, the possibility of a functional difference between bacterial and archaeal NucS cannot be ruled out. Therefore, additional studies are needed to assess whether mycobacterial and archaeal NucS proteins have the same functional requirements.

Our computational studies support an archaeal origin for NucS, as previously suggested^12^, built upon two distinct domains that suffered a complex evolutionary history of transfers and/or losses, including at least two HGT events to Actinobacteria and Deinococcus-Thermus. Indeed, the contribution of horizontal gene transfer in bacterial and archaeal evolution is well-established^33^,^34^. Interestingly, NucS and MutS–MutL systems seem to be present alternatively in different species, with only a few exceptions. In Actinobacteria, it is possible that the acquisition of NucS may have facilitated the subsequent loss of MutS–MutL, as these canonical proteins are so widely conserved across Bacteria.

On the other hand, there are two groups (Halobacteria and some Deinococcus-Thermus) where NucS and MutS–MutL coexist. Notably, in a Halobacteria, *Halobacterium salinarum*, inactivation of *mutS* or *mutL* produced no hypermutability^35^, suggesting that MutS and MutL are redundant to an alternative system that controls spontaneous mutation. This indicates that evolution of these particular species may have opted to keep both systems. The possible interplay between both pathways remains to be elucidated. Surprisingly, NucS and MutS–MutL are apparently absent in some species. Therefore, the possible existence of additional alternative MMR repair pathways, yet to be identified, cannot be discarded.

In conclusion, we propose that MMR is a mechanism that can be either accomplished by either the MutS-L or the NucS pathway. Understanding the mechanisms and pathways that influence genome stability may unveil new strategies to predict and combat the development of drug resistance. In addition, engineered *Mycobacterium* strains lacking this mutation-avoidance pathway, may be valuable tools for evaluating anti-tuberculosis treatments, including new drugs, drug combinations and non-antibiotic based regimes.

## Methods

### Bacterial strains and growth conditions

*M. smegmatis* wild-type strain mc^2^ 155 and its mutant derivatives were grown at 37 °C in Middlebrook 7H9 broth or Middlebrook 7H10 agar (Difco) with 0.5% glycerol and 0.05% Tween 80, and enriched with 10% albumin-dextrose-catalase (Difco). *E. coli* strains were cultured at 37 °C in LB medium. *Streptomyces coelicolor* A3(2) M145 and its derivatives were grown at 30 °C on mannitol–soya (MS) agar.

All primers used in this work are listed in Supplementary Table 6.

### Generation and screening of a *M. smegmatis* insertion library

Transposon ΦMycoMarT7 (ref. 36) was used to obtain a *M. smegmatis* mutant library of 11,000 independent clones. For transduction, *M. smegmatis* mc^2^ 155 cultures were washed, mixed with the phage stock at a multiplicity of infection of 1:10 (37 °C, 3 h) and plated on kanamycin (25 μg ml^−1^). The insertion mutants were isolated and inoculated into 96-well microtiter plates containing Middlebrook 7H9 medium. To identify hypermutators, each mutant was inoculated onto 7H10 agar plates with rifampicin (20 μg ml^−1^). One transposon mutant, producing a high number of rifampicin resistant (Rif-R) colonies, was selected for further characterization. The transposon insertion site in the chromosome was determined by sequencing.

### Generation of a Δ*nucS* knockout mutant in *M. smegmatis*

*M. smegmatis* Δ*nucS* (*MSMEG_4923*, *nucS*_Sm_) in-frame deletion mutant was generated by allelic replacement^37^. Briefly, p2NIL-Δ*nucS*_Sm_, harbouring an in-frame deletion of the target gene, was electroporated into *M. smegmatis* mc^2^ 155. Single-crossover merodiploid clones were isolated and after that, counter-selected to generate the unmarked deletion by a second crossover event. Finally, putative *M. smegmatis* Δ*nucS* colonies were checked by PCR and sequencing. For additional details see Supplementary Methods.

### Complementation of the *M. smegmatis* Δ*nucS* mutant

For complementation, a wild type of the *MSMEG_4923* gene (*nucS*_Sm_) and the wild-type full-length *MT_1321* gene from CDC1551 control strain (*nucS*_TB_), including their own promoter regions, were cloned into the vector pMV361 (ref. 38) and introduced into *M. smegmatis* Δ*nucS* by electroporation. pMV361 is an integrative vector that carries a site-specific integration system derived from the mycobacteriophage L5. It integrates at a single site in the *M. smegmatis* chromosome, the *attB*, which overlaps the 3′ end of the tRNA-glycine gene^39^. Integration at the appropriate site was verified for all constructs by PCR. For additional details see Supplementary Methods.

### Δ*nucS S. coelicolor* knockout mutant and complementation

*S. coelicolor* Δ*nucS* (gene *SCO5388*, *nucS*_Sco_) in-frame deletion mutant was constructed by double crossover recombination using the plasmid pIJ6650 (ref. 40). pIJ-Δ*nucS*_Sco_ (containing the in-frame deletion of the *nucS*_Sco_ gene) was introduced in *S. coelicolor* A3(2) M145 to generate the unmarked deletion of the *nucS*_Sco_ gene. Finally, *S. coelicolor* Δ*nucS* was complemented with a wild-type copy of *nucS*_Sco_ inserted in the *attB* site of the *S. coelicolor* Δ*nucS via* the integrative vector pSET152 (Supplementary Methods).

### Estimation of mutation rates

Fluctuation analyses were used to experimentally address mutation rates. For each experiment, 20 independent cultures of *M. smegmatis* mc^2^ 155 and its derivatives were grown and diluted to inoculate 1,000–10,000 cells per ml into fresh medium. All the cultures were incubated until they reached stationary phase (about 10^9^ cells per ml). Appropriate dilutions were plated on Middlebrook 7H10 medium with or without rifampicin (100 μg ml^−1^) or streptomycin (50 μg ml^−1^). For *S. coelicolor*, 20 independent concentrated spore suspensions (containing ∼10^9^ spores per ml) from each analysed strain were generated on MS agar, and after that, plated on MS agar with or without rifampicin (100 μg ml^−1^) or streptomycin (50 μg ml^−1^). At least three different experiments were performed for each fluctuation analysis in both cases.

The expected number of mutations per culture (m) and 95% confidence intervals were calculated using the maximum likelihood estimator applying the newton.LD.plating and confint.LD.plating functions that account for differences in plating efficiency implemented in the package rSalvador (http://eeeeeric.com/rSalvador/) (Qi Zheng. Rsalvador: an assay. R package version 1.3) for R (www.R-project.org/). Mutation rates (mutations per cell per generation) were then calculated by dividing m by the total number of generations, assumed to be roughly equal to the average final number of cells. Statistical comparisons were carried out by the use of the LRT.LD.plating function of the same package, which accounts for the differences in final number of cells and plating efficiency. Finally, in case of multiple comparisons *P* values were corrected by the Bonferroni method.

### Characterization of the mutational spectrum

The mutational spectra conferred by *M. smegmatis* mc^2^ 155 and its Δ*nucS* derivative were characterized by selecting for resistance to rifampicin and sequencing the rifampicin resistance-determining region in the *rpoB* gene, from independent Rif-R isolates.

### Recombination assays

The recombination assay vectors (pRhomyco series) were generated by cloning two overlapping fragments of the hygromycin-resistance gene (*hyg*), from pRAM^41^, flanking a functional kanamycin-resistance (kan) gene in the pMV361 plasmid (Supplementary Fig. 2). Neither of the fragments of *hyg* conferred resistance to hygromycin on their own. Fragments share a duplicated 517 bp overlapping region, with different degrees of sequence identity (100, 95, 90 or 85%). *M. smegmatis* mc^2^ 155 and its Δ*nucS* derivative were transformed by electroporation with the integrative plasmids pRhomyco 100%, 95%, 90% and 85% designed for the recombination assays and plated on Middlebrook 7H10 containing kanamycin (25 μg μl^−1^). Integration of pRhomyco vectors in the appropriate site (*attB*) of the *M. smegmatis* mc^2^ 155 (wild type and Δ*nucS*) chromosome was verified by PCR.

To measure recombination rates, we analysed the restoration of a functional *hyg* gene that confers Hyg-R by a recombination event between the two overlapping fragments of the *hyg* gene. Sixteen independent overnight cultures from each strain were grown in Middlebrook 7H9 broth plus kanamycin (to prevent the selection of Hyg-R bacteria coming from the early recombination of pRhomyco). Approximately, 10^4^ bacteria were inoculated in plain 7H9 broth (without kanamycin) to allow the recombination events and cultured 24 h at 37 °C with shaking. Cultures were plated on 7H10 plus 50 μg ml^−1^ hygromycin (for recombinant cells count) and in addition, appropriate dilutions were plated on plain 7H10 (for viable cell counts) and incubated for 3–5 days. Total number of Hyg-R colonies was considered to calculate recombination rates. Recombination rates were calculated and analysed as described in estimation of mutation rates.

To validate the recombination assay, we first verified that no Hyg-R colonies were generated by spontaneous mutation, even in the Δ*nucS* derivative. The mutation rate was ≤1 × 10^−10^ for the hypermutable derivative, far below the lowest recombination value (6 × 10^−9^ for WT, 15% non-identical sequences). These results indicate that the Hyg-R colonies from our recombination assay were not generated by spontaneous mutations. Moreover, we verified that recombinant clones express hygromycin resistance and kanamycin susceptibility by picking 20–30 Hyg-R colonies from each strain onto kanamycin plates. In all cases they were Hyg-R and Kan-S. Finally, we randomly isolated 10 independent Hyg-R presumptive recombinant colonies from each construct (80 colonies total) and verified by PCR that a single fragment of the size of the reconstituted *hyg* gene was amplified in all cases.

### NucS purification and biochemical activity

*M. smegmatis nucS* was cloned as a SUMO fusion and expressed in *E. coli* BL21 cells overnight at 16 °C. NucS protein was purified by affinity chromatography using Ni^2+^–NTA agarose column followed by an additional step of anion exchange purification in HiTrap Q HP column. NucS-SUMO fusion was cleaved by Ulp protease to release a native protein. Purified protein was collected and concentrated to perform the activity assays (Supplementary Fig. 1A). In addition, His-tagged *M. smegmatis nucS* was also cloned in pET28b (for *E. coli* expression) and pYUB28b (for *M. smegmatis* expression). His-tagged NucS was also purified by affinity chromatography and anion exchange purification as described before.

For DNA EMSA assays, NucS purified protein (1–16 μM) was incubated with fluorescein-labelled ssDNA (45-mer DNA, 50 nM) or dsDNA (45 bp DNA, 50 nM), in 50 mM Tris–HCl (pH 7.5), 1 mM EDTA, 10% glycerol and 10 μg ml^−1^ BSA for 30 min at 37 °C. Protein–DNA complexes were resolved on 5% polyacrylamide gel in × 0.25 TBE plus 1% glycerol and run under refrigerated conditions (5–10 °C).

For nuclease activity, fluorescein-labelled 36-mer DNA was annealed to a fully complementary opposite strand (control) or carrying a single nucleotide mismatch in the central region of the strand (mismatch substrates). In total, 30 nM dsDNA substrates were incubated with 300 nM of recombinant, tag free NucS in 20 mM Tris–HCl pH 7.5, 6 mM ammonium sulfate, 2 mM MgCl_2_ and 100 mM NaCl, 0.1 mg ml^−1^ BSA and 0.1% TritonX-100. The reactions were carried out at 37 °C for 30 min when 0.5 U Proteinase K was added to each reaction to digest NucS nuclease. Digestion was continued for 30 min at 50 °C. Stop solution was added (95% formamide, 0.09% xylene cyanol), samples were boiled at 95 °C for 10 min and resolved on 7 M urea, 15% polyacrylamide gel in 1 × TBE for 2 h. Mg, Mn and Zn ions, and combinations of these three metals, were tested as cofactors with no cleavage observed.

### Analysis of *nucS* SNPs in *M. tuberculosis* clinical strains

Sequences from the MT_1321 gene product were downloaded from the Ensembl database (http://bacteria.ensembl.org/index.html), *M. tuberculosis* variome resource^42^ and Comas *et al*. data^43^. A total of 1,600 proteins were aligned using MAFFT and the polymorphisms were visualized with Jalview.

### Construction of *nucS* alleles by site-directed mutagenesis

To construct *nucS* alleles, wild-type *nucS*_TB_ gene was PCR amplified and cloned into pUC19 to be used as template for mutagenic PCRs. Single specific mutations were introduced into *nucS*_TB_ by PCR with the suitable mutagenic pairs of primers. Following the PCR reaction, a DpnI digestion was carried out for 4 h at 37 °C. Mutagenized plasmids obtained after transformation into *E. coli* DH5α were verified by DNA sequencing. All the *nucS* alleles were re-cloned into the integrative vector pMV361 to generate a set of complementation plasmids and introduced by transformation into *M. smegmatis* mc^2^ 155 Δ*nucS*. These plasmids integrate at a single site, *attB*, in the *M. smegmatis* chromosome.

To test the efficiency of each *nucS* allele to restore normal mutation rates, the plasmids pMV361 carrying the mutated alleles were integrated in *M. smegmatis* Δ*nucS* chromosome. Integration of each *nucS*_TB_ allele in the appropriate site (*attB*) of the chromosome was verified by PCR.

### Computational analyses of the NucS protein

A summary of all the computational approaches conducted is depicted in Supplementary Fig. 4.

To establish how general the presence of NucS is in the domains on life, we conducted sequence analyses to initially identify NucS proteins (Supplementary Fig. 4). NUCS_MYCTU (*M. tuberculosis* NucS) and NUCS_PYRAB (*P. abyssi* NucS) sequences were used to find homologues. Each full sequence was used as an independent query in pHMMER searches (HMMER3; http://hmmer.org/) against the large database of the concatenated reference proteomes (2016_02 release of 17th February 2016). We next collected sequences excluding the original sequences used to conduct the queries. The remaining sequences were filtered by e-value (removed >0.0001), bit score >50, and length (only those >75% length were kept), and further aligned by MAFFT [ http://mafft.cbrc.jp/alignment/software/]^44^. The alignments were visualized with Belvu [ http://sonnhammer.sbc.su.se/Belvu.html]^45^ to check for quality. We removed the redundancy of the alignment discarding sequences with more than 63% of sequence identity. We next generated Markov profiles trained with the non-redundant multiple sequence alignment (using HHMER3). For each protein, we obtained about the same 400 sequences that would give also partial hits to different species, which suggested a potential existence of different domains in the protein. Further checking of the sequences retrieved the final 370 sequences. As NucS was not identified in eukaryotes or viruses, they were discarded for further analyses.

We constructed a phylogenetic profile of NucS (Supplementary Data 1) and mapped it into the NCBI taxonomic tree (Fig. 5, the newick tree format is available in Supplementary Data 2). The tree was annotated using ggtree (http://www.bioconductor.org/packages/ggtree).

### Characterization of the two NucS regions

To identify potential domains in NucS, we focused on the archaeal *P. abyssi* NucS, whose 3D structure was previously resolved (PDB 2VLD chain B)^11^. This structure is composed by two distinct regions: the N-terminal DNA-binding region (1–114 amino acids) and the C-terminal catalytic region (126–233 amino acids)^11^. Given the absence of structural data for the bacterial NucS protein, the *M. tuberculosis* NucS structure was modelled using the archaeal *P. abyssi* NucS as a template using I-TASSER^16^ and generated a reliable model (Supplementary Fig. 3). Template and model were structurally aligned using the Combinatorial Extension (CE) algorithm^17^, built-in with PyMOL, to identify relevant residues. We used the structure-based alignment between this template and *M. tuberculosis* NucS (NucS_MYCTU) (Supplementary Fig. 3) to determine the different regions for further sequence analyses (N-terminal and C-terminal regions). These analyses were conducted using profile searches as above and confirmed by independent analyses of the PF01939 domain trained with NucS (for details see Supplementary Methods and Supplementary Fig. 4).

We conducted sequence searches of NucS_MYCTU in the PFAM database, where the PF01939 domain (DUF91, automatic) was identified. From the PF01939 domain (539 sequences in 464 species), only 368 entries in 363 species (archaeal and bacterial) gave a match with the full protein (Supplementary Data File 1). The results obtained from the PFAM database searches for the PF01939 domain, are in agreement with the structure-based profiles analyses (see above) confirming the existence of two distinct regions.

Taking into account the domain boundaries established above, we next aligned the 368 full NucS proteins (from PFAM PF01939) to profiles built from the structural alignment corresponding to (i) the whole protein, (ii) the N-terminal region (NT) and (iii) the C-terminal region (CT).

### Sequence analysis of NucS domains

Using the different defined regions by structural bioinformatics, we first conducted sequence searches against the large database. We made non-redundant alignments of the N-terminal region (containing 63 sequences) and the C-terminal region (containing 39 sequences) and built profile HMMs. These profiles were searched using pHMMER^46^ against the large References Proteomes file.

Our analyses identified the C-terminal region in all the original 368 NucS sequences (for details see Supplementary Methods and Supplementary Fig. 4). In addition, the C-terminal was also found in proteins containing a variety of additional protein domains in other prokaryotic species and in few additional eukaryotic sequences. We checked the alignments to confirm that the catalytic residues were conserved, as described in the structure of *P. abyssi*^11^. When a profile with the eukaryotic sequences (excluding any bacterial or archaeal sequences) was generated, we retrieved again the C-terminal regions of NucS above threshold at significant values, but we did not retrieve any other eukaryotic sequences at reliable values. On the other hand, the N-terminal region was found mainly in bacterial and archaeal NucS, but also in two archaeal proteins annotated as topoisomerases. A pHMMER search of the regions of these sequences yielded the N-terminal of NucS.

### Phylogenetic analyses

We conducted Maximum Likelihood (ML) phylogenetic analyses using RAxML^47^ to construct phylogenetic trees that could explain the phylogenetic profile (Supplementary Fig. 4).

To run phylogenetic analyses, NucS sequences from the PF01939 domain were used, but aligned to their corresponding structure-based regions. Sequences found in our searches with independent regions were also included. Out of 539, 368 unique sequences aligned with the full NucS structure-based profile (Supplementary Fig. 5, Supplementary Data 3), 422 sequences containing the C-terminal region (NucS-CT) (Supplementary Fig. 6), 370 sequences containing the N-terminal region (NucS-NT) (Supplementary Fig. 7) were run.

Sequences were aligned to the structure-based profiles, and the alignments were subjected to ML search and 1,500 bootstrap replicates using RAXML^47^. All free model parameters were estimated by RAxML where we used a GAMMA model of rate heterogeneity and ML estimate of alpha-parameter. The most likely selected model was LG^48^. For accuracy and focus on our alignment, we run the sequential version of RAxML creating various sets of starting trees (10 by manual inferring different starting trees and 10 as automatically inferred by the programme). The best setting (the closest likelihood to zero) was used for further calculations.

To run phylogenetic analyses, sequences from the PFAM PF01939 domain were used, but aligned to their corresponding region. For details of the particular regions for each domain see Supplementary Data 4. Sequences found in our searches with NT or CT regions outside NucS were also included. Phylogenetic trees were visualized with iTOL (http://itol.embl.de)^49^. The raw trees corresponding to both CT and NT regions (Supplementary Figs 6 and 7) are available as Supplementary Data 5 and 6 respectively.

### Data availability

The authors declare that all data supporting the findings of this study are available within the paper and its Supplementary Information files or from the authors upon reasonable request.

## Additional information

**How to cite this article:** Castañeda-García, A. *et al*. A non-canonical mismatch repair pathway in prokaryotes. *Nat. Commun.* **8,** 14246 doi: 10.1038/ncomms14246 (2017).

**Publisher's note:** Springer Nature remains neutral with regard to jurisdictional claims in published maps and institutional affiliations.

## Supplementary Material

Supplementary Information Supplementary Figures, Supplementary Tables, Supplementary Methods, and Supplementary References

Supplementary Dataset 1 Taxonomic distribution of NucS and MutS-MutL in reference proteomes (Big excel data file).

Supplementary Dataset 2 Taxonomic tree for phylogenetic profiling in newick format. Raw NCBI profiling tree from archaeal and bacterial species extracted from NCBI in newick format, corresponding to Figure 5.

Supplementary Dataset 3 Phylogenetic tree of full NucS in newick format. Raw Maximum Likelihood and bootstrap phylogenetic tree corresponding to Supplementary Figure 5 (full NucS) in newick format.

Supplementary Dataset 4 Detailed information for phylogenetic trees of NucS regions. Sequence details of NucS-CT and NucS-NT labels on Supplementary Figures 6-7 trees (Excel file).

Supplementary Dataset 5 Phylogenetic tree of C-terminal NucS in newick format. Raw Maximum Likelihood and bootstrap phylogenetic tree corresponding to Supplementary Figure 6 (NucS-CT region) in newick format.

Supplementary Dataset 6 Phylogenetic tree of N-terminal NucS in newick format. Raw Maximum Likelihood and bootstrap phylogenetic tree corresponding to Supplementary figure 7 (NucS-NT region) in newick format.

## References

[b1] Friedberg E. . in DNA Repair and Mutagenesis 2nd edn American Society of Microbiology (2006).

[b2] Eisen J. A. & Hanawalt P. C. A phylogenomic study of DNA repair genes, proteins, and processes. Mutat. Res. 435, 171–213 (1999). 10606811 10.1016/s0921-8777(99)00050-6 PMC3158673

[b3] Iyer R. R., Pluciennik A., Burdett V. & Modrich P. L. DNA mismatch repair: functions and mechanisms. Chem. Rev. 106, 302–323 (2006). 16464007 10.1021/cr0404794

[b4] Matic I., Rayssiguier C. & Radman M. Interspecies gene exchange in bacteria: the role of SOS and mismatch repair systems in evolution of species. Cell 80, 507–515 (1995). 7859291 10.1016/0092-8674(95)90501-4

[b5] Sachadyn P. Conservation and diversity of MutS proteins. Mutat. Res. 694, 20–30 (2010). 20833188 10.1016/j.mrfmmm.2010.08.009

[b6] Mizrahi V. & Andersen S. J. DNA repair in *Mycobacterium tuberculosis*. What have we learnt from the genome sequence? Mol. Microbiol. 29, 1331–1339 (1998). 9781872 10.1046/j.1365-2958.1998.01038.x

[b7] Banasik M. & Sachadyn P. Conserved motifs of MutL proteins. Mutat. Res. 769, 69–79 (2014). 25771726 10.1016/j.mrfmmm.2014.07.006

[b8] Springer B. . Lack of mismatch correction facilitates genome evolution in mycobacteria. Mol. Microbiol. 53, 1601–1609 (2004). 15341642 10.1111/j.1365-2958.2004.04231.x

[b9] Ford C. B. . Use of whole genome sequencing to estimate the mutation rate of *Mycobacterium tuberculosis* during latent infection. Nat. Genet. 43, 482–486 (2011). 21516081 10.1038/ng.811 PMC3101871

[b10] Kucukyildirim S. . The rate and spectrum of spontaneous mutations in *Mycobacterium smegmatis*, a bacterium naturally devoid of the post-replicative mismatch repair pathway. G3 (Bethesda) 6, 2157–2163 (2016). 27194804 10.1534/g3.116.030130 PMC4938668

[b11] Ren B. . Structure and function of a novel endonuclease acting on branched DNA substrates. EMBO J. 28, 2479*-*–2489 (2009). 19609302 10.1038/emboj.2009.192 PMC2735178

[b12] Ishino S. . Identification of a mismatch-specific endonuclease in hyperthermophilic Archaea. Nucleic Acids Res. 44, 2977–2986 (2016). 27001046 10.1093/nar/gkw153 PMC4838380

[b13] Wagner R. . Involvement of *Escherichia coli* mismatch repair in DNA replication and recombination. Cold Spring Harb. Symp. Quant. Biol. 49, 611–615 (1984). 6397316 10.1101/sqb.1984.049.01.069

[b14] Nakae S. . Structure of the EndoMS-DNA complex as mismatch restriction endonuclease. Structure 24, 1960–1971 (2016). 27773688 10.1016/j.str.2016.09.005

[b15] Gross M. D. & Siegel E. C. Incidence of mutator strains in *Escherichia coli* and coliforms in nature. Mutat. Res. 91, 107–110 (1981). 7019693 10.1016/0165-7992(81)90081-6

[b16] LeClerc J. E., Li B., Payne W. L. & Cebula T. A. High mutation frequencies among *Escherichia coli* and Salmonella pathogens. Science 274, 1208–1211 (1996). 8895473 10.1126/science.274.5290.1208

[b17] Matic I. . Highly variable mutation rates in commensal and pathogenic *Escherichia coli*. Science 277, 1833–1834 (1997). 9324769 10.1126/science.277.5333.1833

[b18] Oliver A., Canton R., Campo P., Baquero F. & Blazquez J. High frequency of hypermutable *Pseudomonas aeruginosa* in cystic fibrosis lung infection. Science 288, 1251–1254 (2000). 10818002 10.1126/science.288.5469.1251

[b19] Picard B. . Mutator natural *Escherichia coli* isolates have an unusual virulence phenotype. Infect. Immun. 69, 9–14 (2001). 11119483 10.1128/IAI.69.1.9-14.2001 PMC97849

[b20] Giraud A., Matic I., Radman M., Fons M. & Taddei F. Mutator bacteria as a risk factor in treatment of infectious diseases. Antimicrob. Agents Chemother. 46, 863–865 (2002). 11850274 10.1128/AAC.46.3.863-865.2002 PMC127494

[b21] Macia M. D. . Hypermutation is a key factor in development of multiple-antimicrobial resistance in *Pseudomonas aeruginosa* strains causing chronic lung infections. Antimicrob. Agents Chemother. 49, 3382–3386 (2005). 16048951 10.1128/AAC.49.8.3382-3386.2005 PMC1196247

[b22] Muller B., Borrell S., Rose G. & Gagneux S. The heterogeneous evolution of multidrug-resistant *Mycobacterium tuberculosis*. Trends Genet. 29, 160–169 (2013). 23245857 10.1016/j.tig.2012.11.005 PMC3594559

[b23] Ford C. B. . *Mycobacterium tuberculosis* mutation rate estimates from different lineages predict substantial differences in the emergence of drug-resistant tuberculosis. Nat. Genet. 45, 784–790 (2013). 23749189 10.1038/ng.2656 PMC3777616

[b24] Cox E. C. Bacterial mutator genes and the control of spontaneous mutation. Annu. Rev. Genet. 10, 135–156 (1976). 797306 10.1146/annurev.ge.10.120176.001031

[b25] Lee H., Popodi E., Tang H. & Foster P. L. Rate and molecular spectrum of spontaneous mutations in the bacterium *Escherichia coli* as determined by whole-genome sequencing. Proc. Natl Acad. Sci. USA 109, E2774–E2783 (2012). 22991466 10.1073/pnas.1210309109 PMC3478608

[b26] Garibyan L. . Use of the rpoB gene to determine the specificity of base substitution mutations on the *Escherichia coli* chromosome. DNA Repair 2, 593–608 (2003). 12713816 10.1016/s1568-7864(03)00024-7

[b27] Tham K. C. . Mismatch repair inhibits homeologous recombination via coordinated directional unwinding of trapped DNA structures. Mol. Cell 51, 326–337 (2013). 23932715 10.1016/j.molcel.2013.07.008 PMC3781583

[b28] Feinstein S. I. & Low K. B. Hyper-recombining recipient strains in bacterial conjugation. Genetics 113, 13–33 (1986). 3519362 10.1093/genetics/113.1.13 PMC1202792

[b29] Spies M. & Fishel R. Mismatch repair during homologous and homeologous recombination. Cold Spring Harb. Perspect. Biol. 7, a022657 (2015). 25731766 10.1101/cshperspect.a022657 PMC4355274

[b30] Rock J. M. . DNA replication fidelity in *Mycobacterium tuberculosis* is mediated by an ancestral prokaryotic proofreader. Nat. Genet. 47, 677–681 (2015). 25894501 10.1038/ng.3269 PMC4449270

[b31] Ebrahimi-Rad M. . Mutations in putative mutator genes of *Mycobacterium tuberculosis* strains of the W-Beijing family. Emerg. Infect. Dis. 9, 838–845 (2003). 12890325 10.3201/eid0907.020803 PMC3023437

[b32] Dos Vultos T., Blazquez J., Rauzier J., Matic I. & Gicquel B. Identification of Nudix hydrolase family members with an antimutator role in *Mycobacterium tuberculosis* and *Mycobacterium smegmatis*. J. Bacteriol. 188, 3159–3161 (2006). 16585780 10.1128/JB.188.8.3159-3161.2006 PMC1446978

[b33] Dagan T., Artzy-Randrup Y. & Martin W. Modular networks and cumulative impact of lateral transfer in prokaryote genome evolution. Proc. Natl Acad. Sci. USA 105, 10039–10044 (2008). 18632554 10.1073/pnas.0800679105 PMC2474566

[b34] Gophna U., Charlebois R. L. & Doolittle W. F. Have archaeal genes contributed to bacterial virulence? Trends Microbiol. 12, 213–219 (2004). 15120140 10.1016/j.tim.2004.03.002

[b35] Busch C. R. & DiRuggiero J. MutS and MutL are dispensable for maintenance of the genomic mutation rate in the halophilic archaeon *Halobacterium salinarum* NRC-1. PLoS ONE 5, e9045 (2010). 20140215 10.1371/journal.pone.0009045 PMC2816208

[b36] Sassetti C. M., Boyd D. H. & Rubin E. J. Comprehensive identification of conditionally essential genes in mycobacteria. Proc. Natl Acad. Sci. USA 98, 12712–12717 (2001). 11606763 10.1073/pnas.231275498 PMC60119

[b37] Parish T. & Stoker N. G. Use of a flexible cassette method to generate a double unmarked *Mycobacterium tuberculosis* tlyA plcABC mutant by gene replacement. Microbiology 146, 1969–1975 (2000). 10931901 10.1099/00221287-146-8-1969

[b38] Stover C. K. . New use of BCG for recombinant vaccines. Nature 351, 456–460 (1991). 1904554 10.1038/351456a0

[b39] Lee M. H., Pascopella L., Jacobs W. R. Jr & Hatfull G. F. Site-specific integration of mycobacteriophage L5: integration-proficient vectors for *Mycobacterium smegmatis*, *Mycobacterium tuberculosis*, and bacille Calmette-Guerin. Proc. Natl Acad. Sci. USA 88, 3111–3115 (1991). 1901654 10.1073/pnas.88.8.3111 PMC51395

[b40] Paget M. S. . Mutational analysis of RsrA, a zinc-binding anti-sigma factor with a thiol-disulphide redox switch. Mol. Microbiol. 39, 1036–1047 (2001). 11251822 10.1046/j.1365-2958.2001.02298.x

[b41] Hinds J. . Enhanced gene replacement in mycobacteria. Microbiology 145, 519–527 (1999). 10217485 10.1099/13500872-145-3-519

[b42] Joshi K. R., Dhiman H. & Scaria V. tbvar: a comprehensive genome variation resource for *Mycobacterium tuberculosis*. Database (Oxford) 2014, bat083 (2014). 24408216 10.1093/database/bat083 PMC3885892

[b43] Comas I. . Out-of-Africa migration and Neolithic coexpansion of *Mycobacterium tuberculosis* with modern humans. Nat. Genet. 45, 1176–1182 (2013). 23995134 10.1038/ng.2744 PMC3800747

[b44] Katoh K., Misawa K., Kuma K. & Miyata T. MAFFT: a novel method for rapid multiple sequence alignment based on fast Fourier transform. Nucl. Acids Res. 30, 3059–3066 (2002). 12136088 10.1093/nar/gkf436 PMC135756

[b45] Sonnhammer E. L. & Hollich V. Scoredist: a simple and robust protein sequence distance estimator. BMC Bioinform. 6, 108 (2005). 10.1186/1471-2105-6-108 PMC1131889 15857510

[b46] Eddy S. R. A new generation of homology search tools based on probabilistic inference. Genome Inform. 23, 205–211 (2009). 20180275

[b47] Stamatakis A. RAxML-VI-HPC: maximum likelihood-based phylogenetic analyses with thousands of taxa and mixed models. Bioinformatics 22, 2688–2690 (2006). 16928733 10.1093/bioinformatics/btl446

[b48] Le S. Q. & Gascuel O. An improved general amino acid replacement matrix. Mol. Biol. Evol. 25, 1307–1320 (2008). 18367465 10.1093/molbev/msn067

[b49] Letunic I. & Bork P. Interactive tree of life (iTOL) v3: an online tool for the display and annotation of phylogenetic and other trees. Nucleic ACIDS Res. 44, W242–W245 (2016). 27095192 10.1093/nar/gkw290 PMC4987883

[b50] Hug L. A. . A new view of the tree of life. Nat. Microbiol. 1, 16048 (2016). 27572647 10.1038/nmicrobiol.2016.48

