# Supplementary material for: A non-canonical mismatch repair pathway in prokaryotes
Source: Nat Commun. 2017 Jan 27;8:14246. doi: 10.1038/ncomms14246 (PMC5290159; doi:10.1038/ncomms14246)
Supplement: Supplementary Information — Supplementary Figures, Supplementary Tables, Supplementary Methods, and Supplementary References [file ncomms14246-s1.pdf]

Supplementary Information

Supplementary Figures.

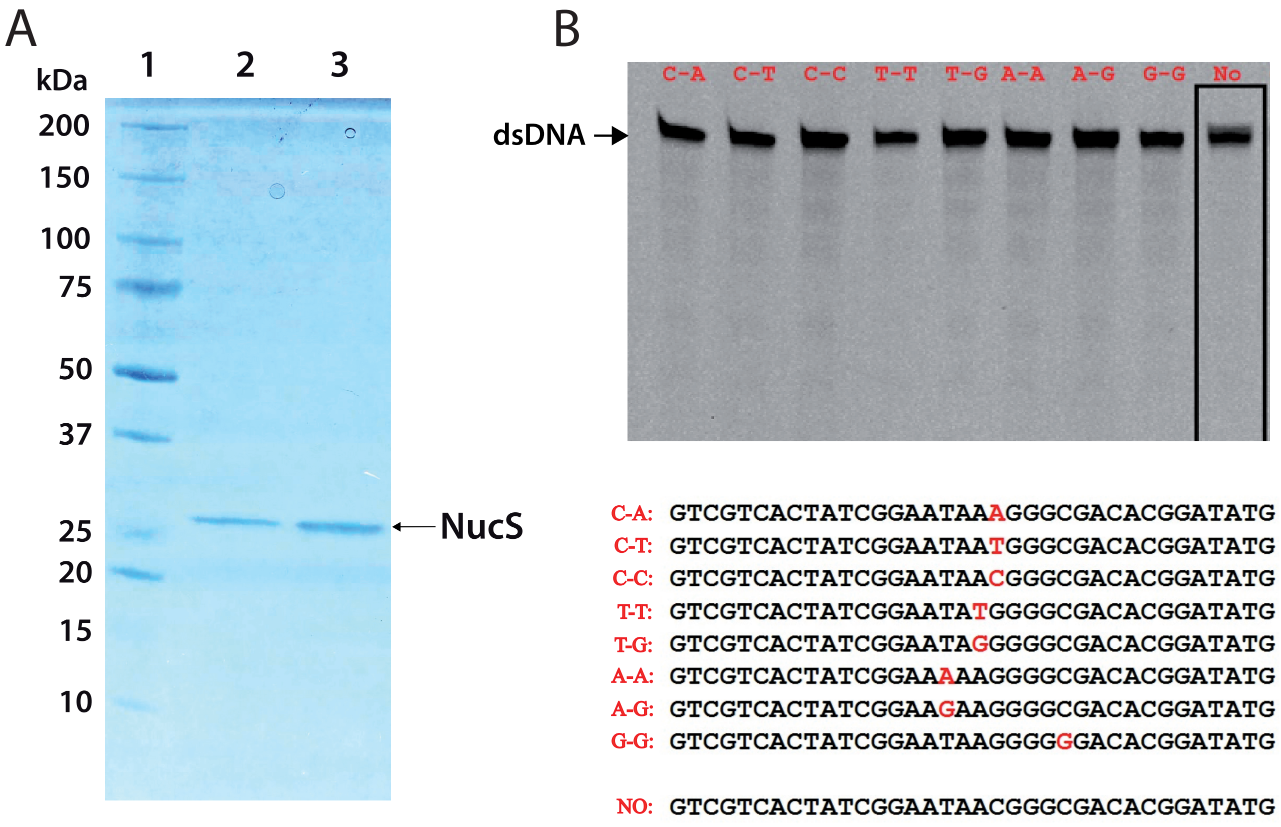

Supplementary Figure 1. Purified mycobacterial NucS does not cleave mismatch DNA substrates in vitro. A) Native *M. smegmatis* NucS protein purification. Recombinant NucS protein was expressed in *E. coli*, purified and concentrated, as described (see Methods). Native NucS protein was analysed in a SDS-PAGE gel and stained with Coomassie Brillant Blue. Lane 1, molecular marker; Lane 2: purified native *M. smegmatis* NucS; Lane 3: concentrated *M. smegmatis* NucS protein (used for EMSA and nuclease assays). B) Double stranded DNA (30 nM) with or without the indicated internal mismatches, was incubated with 300 nM NucS. Sequence consensus is shown at the bottom and mismatch positions are indicated in red. No significant cleavage activity was observed with the shown 36-mers or with longer 75-mer (not displayed here) mismatch substrates.

A

```
100% tgatcaagctgttcggcgagcactggtgcggtccggagagcctcgcgtcggagtcggaggcgtacgcggtcctg74
 95% tgatcaagctgttcggGgagcactggtgcggtccCgagagcctcgcgtcggagtcCgaggcgtacgcggtcctC74
 90% tgatcaagctCttcggGgaAcactggtgcggCccCgagagcctGgcgtcggagtcCgaggcgtacgcCgtcctC74
 85% tgatcaagctCttcggGgaAcactggtgcggCccCgaAagcctGgcgtcCgagtcCgaggCtacgcCgtcctC74

100% gcggacgccccggtgccggtgccccgcctcctcggccgcggcgagctgcggcccggcaccggagcctggccgtg148
 95% gcggacgccccggtgccggtCcccgcctcctcggccgGggcgagctgcggcccggcacGgagcctggccgtg148
 90% gcggacgccccCgtgccggtCcccgcctGctcggccgGggcgagctgcggccGggcacGgagcctggccCtg148
 85% gcggacgcGccCgtgccggtCcccgcctGctcggccgGggcgagctCcggccGggcacGgagcGtggccCtg148

100% gccctacctggtgatgagccggatgaccggcaccacctggcggtccgcgatggacggcacgaccgaccggaacg222
 95% gccGtacctggtgatgagccgCatgaccggcaccacctggcgCtccgcgatggacggcacgacGgaccggaacg222
 90% gccGtacctggtCatgagccgCatgaccggGaccacctggcgCtccgcgatggacggGacgacGgaccggaacg222
 85% gccGtacctggtCatgagccgCatgaccggGaccacGtggcgCtccgcCatggacggGacgacGgaccgCaacg222

100% cgctgctcgccctggcccgcgaactcggccgggtgctcggccggctgcacagggtgccgctgaccgggaacacc296
 95% cgctgctcgccctCgcccgcgaactcggccgggtgctGgccggctgcacagggtgccgctCaccgggaacacc296
 90% cCctgctcgccctCgcccgcgaactGggccgggtgctGgccggctCcacagggtgccgctCaccgggaacacG296
 85% cCctgctGgccctCgcccgcgaGctGggccgCgtgctGgccggctCcacagggtCccgctCaccggCaacacG296

100% gtgctcacccccccattccgaggtcttcccggaactgctgcgggaacgccgcgcggcgaccgtcgaggaccaccg370
 95% gtgctcaccccGcattccgaggtcttcccggaactCctgcgggaacgccgcgcggcgacGgtcgaggaccaccg370
 90% gtgctcaccccGcattccgaggtGttcccggaactCctgcgggaacgGcgcgcggcgacGgtcgaggaccaccg370
 85% gtgctGaccccGcattcGgaggtGttcccCgaactCctgcgCgaacgGcgcgcCgcgacGgtcgaAgaccaccg370

100% cgggtggggctacctctcgccccggctgctggaccgcctggaggactggctgccggacgtggacacgctgctgg444
 95% cgggtggggGtacctctcgccccggctgctCgaccgcctggaggactggctgccCgacgtggacacgctgctCg444
 90% GgggtggggGtacctctcgccGcggctgctCgaccgcctCgaggactggctgccCgacgtggacacCctgctCg444
 85% GgggtggggGtacctGtcgccGcggctgctCgaccgcctCgaggactggctCccCgacgtCgacacCctgctCg444

100% ccggccgcgaaccccggttcgtccacggcgacctgcacgggaccaacatcttcgtggacctggccgcgaccga 517
 95% ccggccgcgaaccccgCttcgtccacggcgacctCcacgggaccaacatcttcgtCgacctggccgcgacGga 517
 90% ccggccgGgaaccccgCttcgtccacggGgacctCcacgggacGaacatcttcgtCgacctggcGgcgacGga 517
 85% ccggccgGgaaccccgCttcgtGcacggGgacctCcacggCacGaacatcttcgtCgacctCgcGgcgacGga 517
```

B

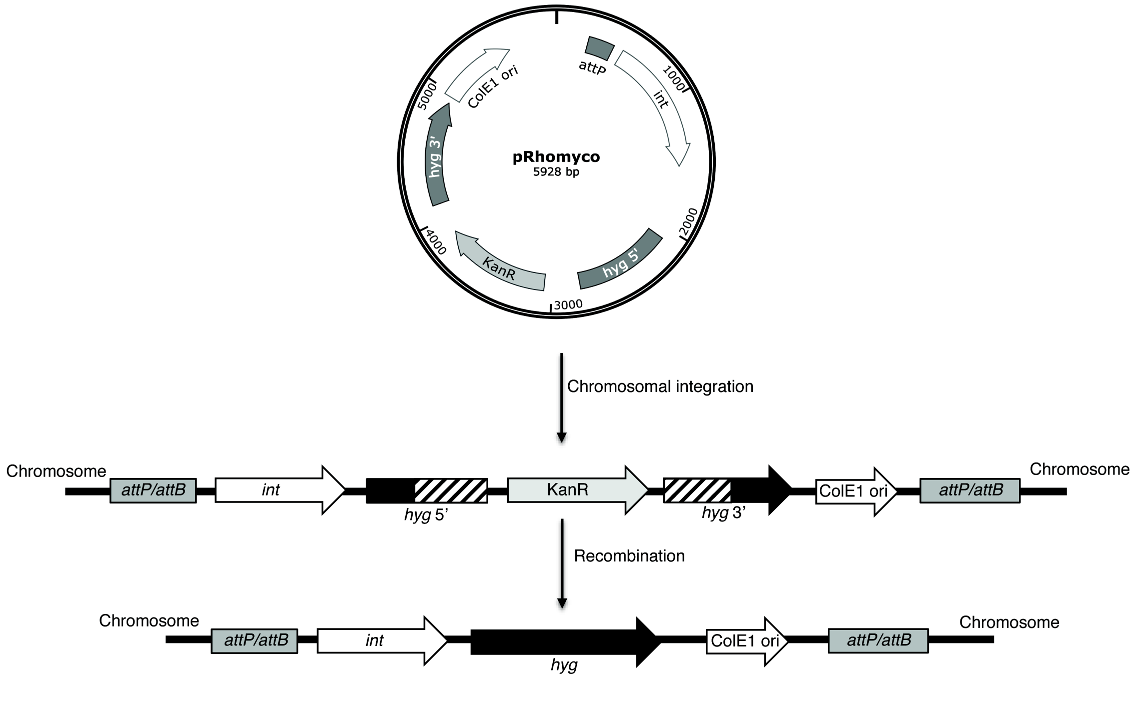

Supplementary Figure 2. Tools for measuring recombination rates.

A) Alignment of the 517 bp overlapping sequences, with 100%, 95%, 90% and 85% identities, shared by *hyg* 5´ and *hyg* 3´ regions, used to construct the different pRhomyco versions. Changes

introduced in the original sequence are highlighted. B) Cartoon of plasmid pRhomyco before and after recombination. *hyg* 5’ and *hyg* 3’ are truncated alleles of the *hyg* gene carrying a 3´-terminal or 5´-terminal deletion, respectively. Both alleles overlap 517 bp (striped regions) and are separated by a 1,200 bp region containing *aph3* gene (Kan-R). Four versions of pRhomyco were generated carrying *hyg* 5’ alleles 100%, 95%, 90% or 85% identical, in its overlapping region, to the *hyg* 3’. Plasmids integrate in the chromosome of *M. smegmatis* mc^2 155 and its Δ*nucS* derivative. Site-specific recombination between the *attP* from the plasmid and the unique bacterial *attB* is promoted by the plasmid-encoded integrase.

A

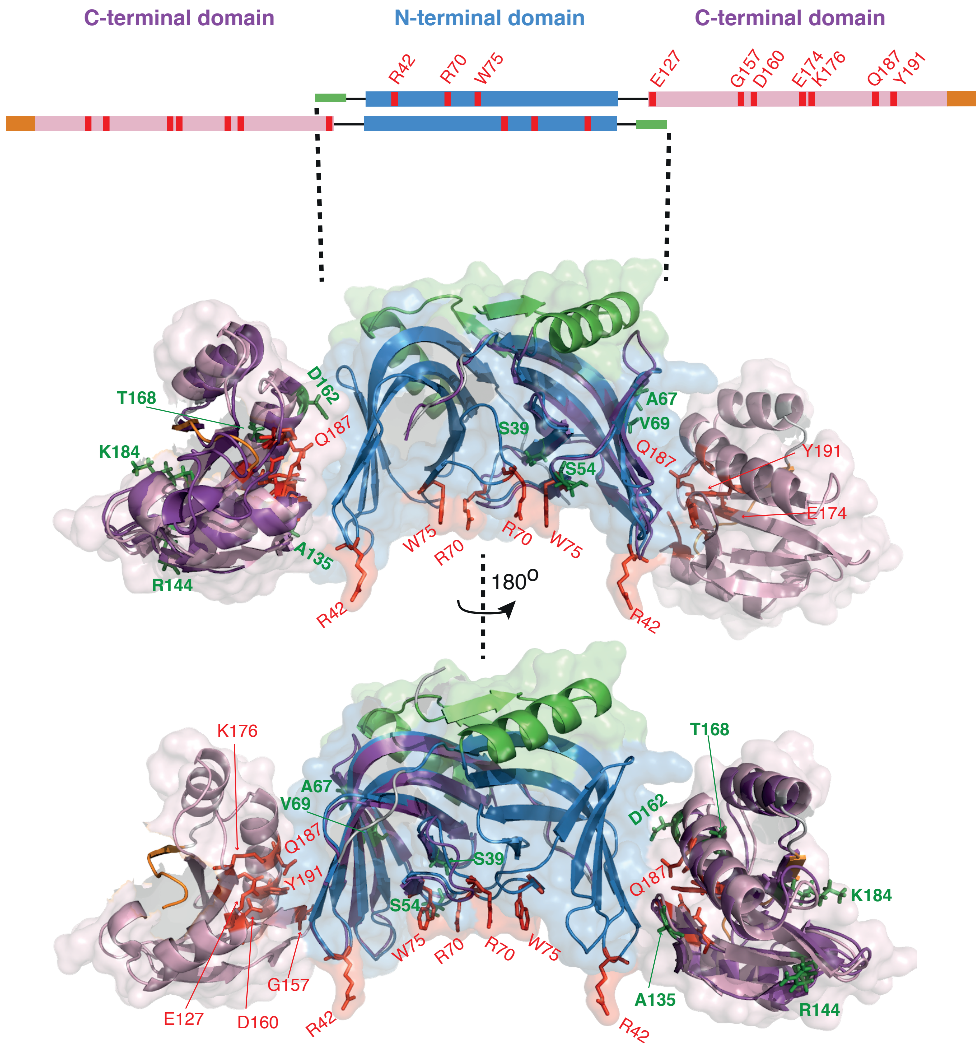

B

```
2VLD_Paby       25 HGGVVTIFARCKVHYEGRAKSELGEGDRIIIIKPDGSFLIHQN-KKREPVNWQPPGSKVT
NucS_Mycsme      1 M---RLVIAQCTVDYVGRLTAHLPSARRLLLFKADGSVSVHADDRAYKPLNWMSPPCWVT
NucS_Myctu       1 MSRVRLVIAQCTVDYIGRLTAHLPSARRLLLFKADGSVSVHADDRAYKPLNWMSPPCWLT
                           * * * * **    *    *    * *** R *       * ** I*    *

2VLD_Paby       84 FK--E--NSmISIRRRPYERLEVEIIEPYSLVVFLAEDYEELaltgSEAEmANLIFENPR
NucS_Mycsme     58 EQDTETGVALWVVENKTGEQLRITVEDIEHDSHHELGVDPGLVKDGVEAHLQALLAEHVE
NucS_Myctu      61 EESGGQ-APVWVVENKAGEQLRITIEGIEHDSSHELGVDPGLVKDGVEAHLQALLAEHIQ
                          S A        * *                    *   * **    *  *

2VLD_Paby      140 VIEEGFKPIYREKPIRHGIVDVMGVDKDGNIVVLELKRRKADLHAVSQLKRYVDSLKEEY
NucS_Mycsme    118 LLGAGYTLVRREYPTPIGPVDLLCRDELGRSVAVEIKR-RGEIDGVEQLTRYLELLNRDS
NucS_Myctu     120 LLGEGYTLVRREYMTAIGPVDLLCRDERGGSVAVEIKR-RGEIDGVEQLTRYLELLNRDS
                       *     **   S * **   S*  *  *  * **     H * **A**   *

2VLD_Paby      200 -GENVRGILVAPSLTEGAKKLLEKEGLEFRKLEPP 233
NucS_Mycsme    177 LLAPVAGVFAAQQIKPQARTLATDRGIRCVTLDYD 211
NucS_Myctu     179 VLAPVKGVFAAQQIKPQARILATDRGIRCLTLDYD 213
                       *E*   *      *  *    *     *
```

Supplementary Figure 3. Domain characterization of *M. tuberculosis* NucS and polymorphic residues. A) The upper part is a schematic representation of the homodimeric structure of *P. abyssi* NucS (2VDL) [1]. The two distinct domains of NucS, N-terminal and C-terminal, are in blue and

pink, respectively. The first residues (1-25) of the NucS *P. abyssi* N-terminal domain, missing in the *M. tuberculosis* model, are depicted as green cartoon. The putative β-clamp binding motif in *P. abyssi* NucS is shown in orange. The important catalytic and DNA binding sites are depicted over the *P. abyssi* structure as vertical red bars with the residues numbered above. Back and front views of the structural superimposition of the homodimeric resolved structure of NucS from *P. abyssi* [1] and the *M. tuberculosis* model (purple) is presented below the schema (colour code as described for the upper scheme). The important catalytic and DNA binding sites are depicted as red sticks and the residues where polymorphisms described in this work have been detected are shown as green sticks over the structural imposition. B) Multiple structure-based alignment of NucS from *P. abyssi* (2VDL), *M. smegmatis* mc^2 155 and *M. tuberculosis* CDC155. Colour code is the same as in panel A. The amino acid change of each naturally occurring *M. tuberculosis* polymorphism is depicted in green. Important catalytic and DNA binding residues are in red. Grey low-case indicates regions without structural information; magenta "m" indicates Seleno-Met modifications in the structure of *P. abyssi*. Residues of the putative β-clamp binding motif in *P. abyssi* NucS are shown in orange. Only regions that align among the three proteins are shown. Asterisks indicate identical residues in the three aligned sequences.

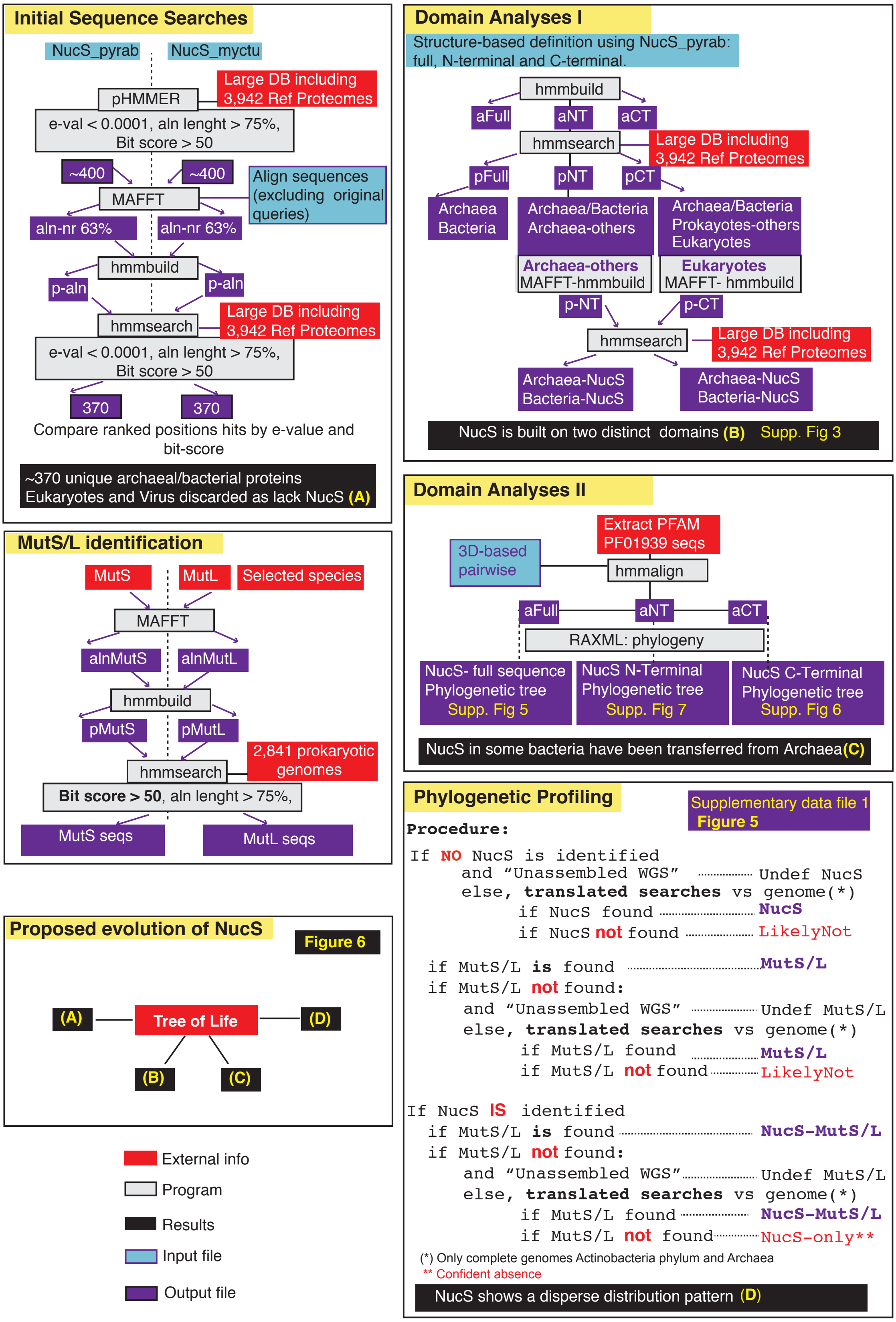

Supplementary Figure 4. Computational procedures to analyse NucS distribution and evolution. Each panel depicts a different protocol. Yellow fonts indicate figures and/or tables. Yellow uppercase letters are pieces of evidence used in the model presented in Fig. 6 (main text).

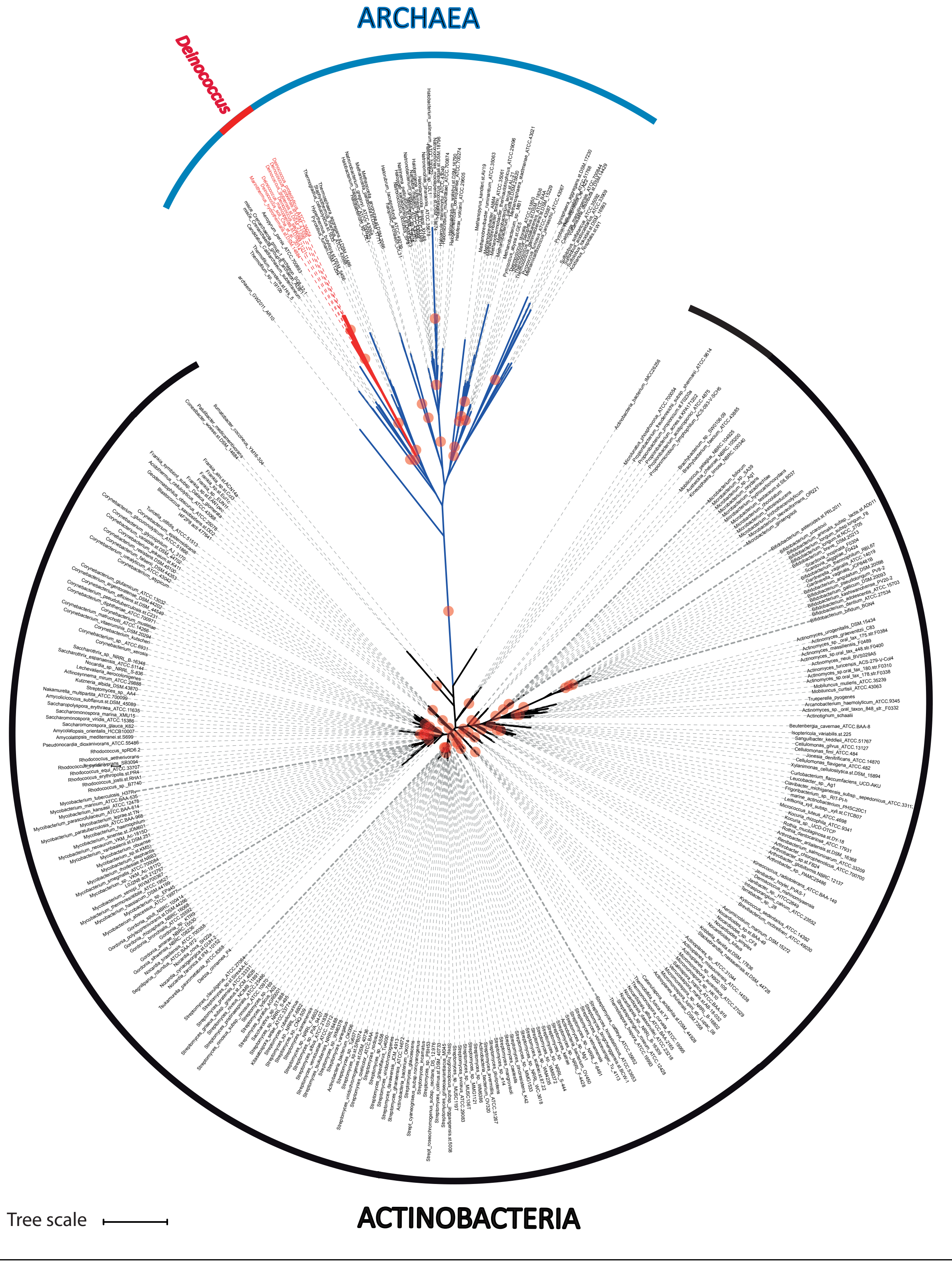

Supplementary Figure 5. Phylogenetic analyses of NucS. Unrooted ML tree of full NucS sequences from the PFAM PF01939. Black branches are Actinobacteria; blue branches are Archaea; red branches are species from the Deinococcus-Thermus group. Labels indicate species name. Red circles represent >80% bootstrap in 1,500 replicates.

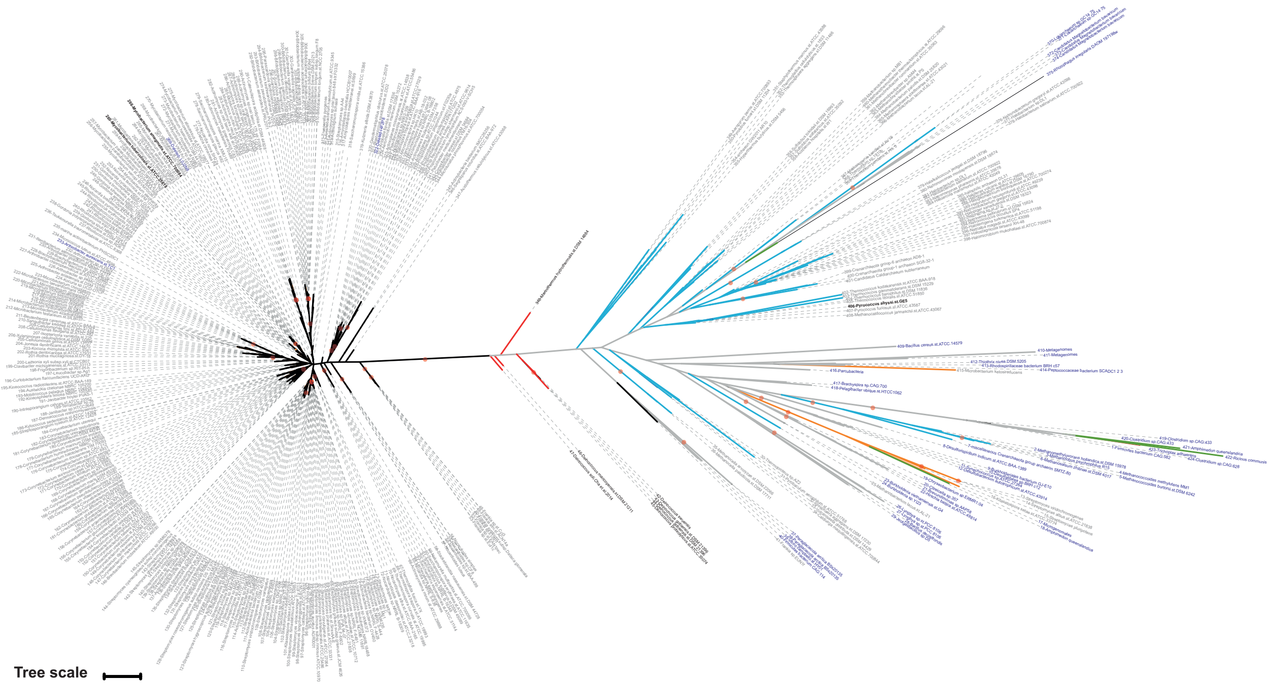

Supplementary Figure 6. Phylogenetic analyses of the NucS C-terminal (CT) region. Unrooted ML tree of CT sequences from the PFAM PF01939 domain. Grey font indicates that this domain is in NucS and blue font that is found outside NucS. Black branches, Actinobacteria; red Deinococcus-Thermus; grey, other Bacteria; light blue, Archaea; green, eukaryotic sequences. Bold fonts indicate the main species used in this study. Labels indicate species name. The circles represent >80% bootstrap in 1,500 replicates.

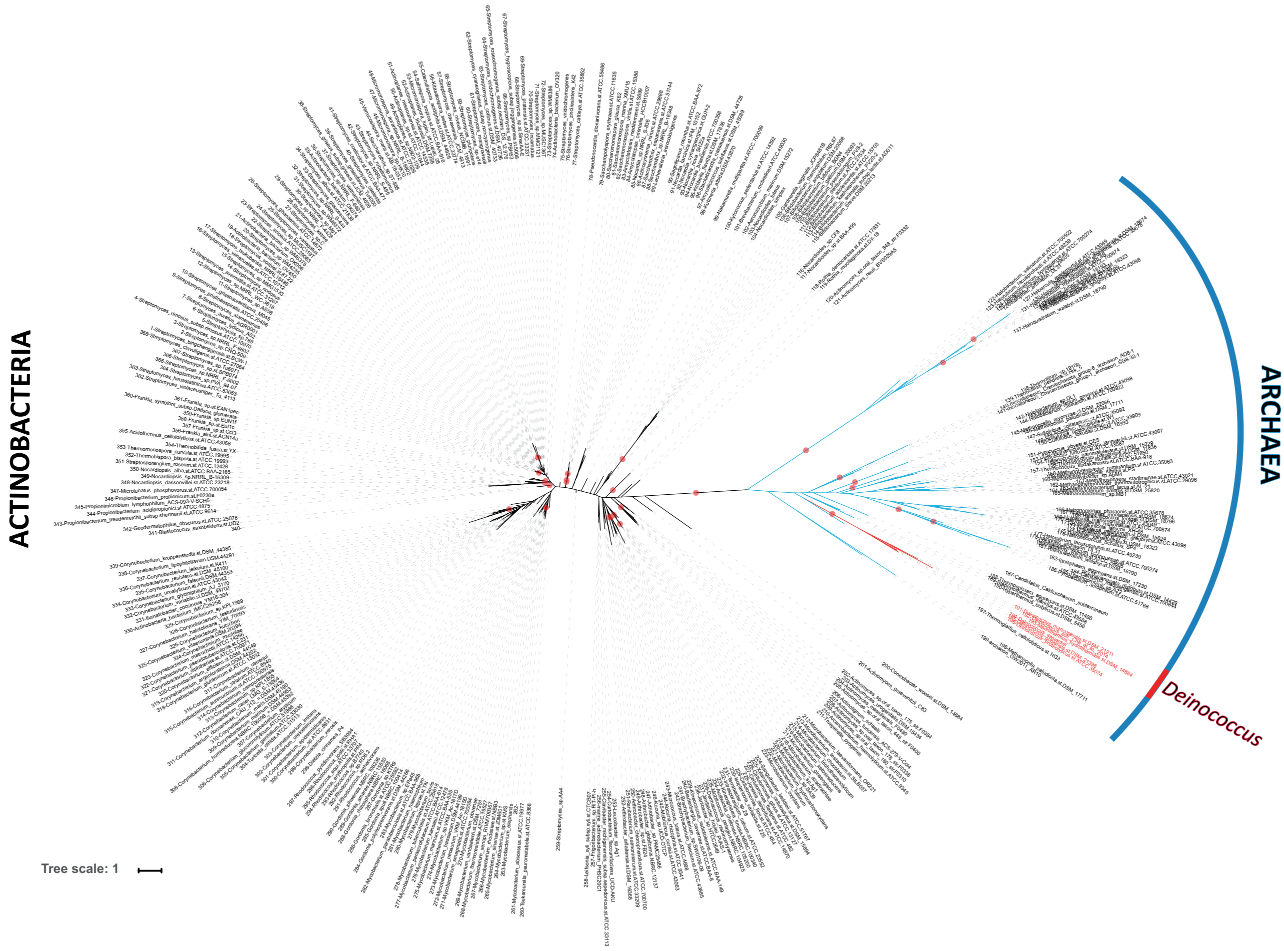

Supplementary Figure 7. Phylogenetic analyses of NucS N-terminal (NT) region. Unrooted ML tree of NT sequences from the PFAM PF01939 domain. Black branches, Actinobacteria; red Deinococcus-Thermus; light blue, Archaea. Labels indicate species name. The circles represent >80% bootstrap in 1,500 replicates.

Supplementary Tables

Supplementary Table 1. Mutation rates of *M. smegmatis* and its Δ*nucS* derivatives. Rates of spontaneous mutations conferring rifampicin (Rif-R) and streptomycin resistance (Str-R) of *M. smegmatis* mc^2 155 (WT), *M. smegmatis* Δ*nucS* and *M. smegmatis* Δ*nucS* complemented with the wild-type *nucS* from *M. smegmatis* mc^2 155 (*nucS*$_{\mathrm{Sm}}$). Fold change indicates the increase in mutation rate with respect to the strain *M. smegmatis* mc^2 155 (set to 1). Mut Rate: mutation rate (mutations per cell per generation).

Strain	Genotype	Mut Rate Rif	95% CI	Fold	Mut Rate Str	95% CI	Fold
mc^2 155	WT	$2.07x10^{-9}$	$1.47\text{-}2.74x10^{-9}$	1	$5.58x10^{-10}$	$2.25\text{-}11.1x10^{-10}$	1
ΔnucS	*ΔnucS*	$3.11x10^{-7}$	$2.81\text{-}3.39x10^{-7}$	150.2	$4.82x10^{-8}$	$3.53\text{-}6.17x10^{-8}$	86.3
ΔnucS/nucS$_{\mathrm{Sm}}$	Complemented	$3.02x10^{-9}$	$2.2\text{-}3.97x10^{-9}$	1.46	$1.43x10^{-9}$	$0.77\text{-}2.33x10^{-9}$	2.56

Supplementary Table 2. Mutational spectrum of *M. smegmatis* mc² 155 and its Δ*nucS* derivative. A) Spontaneous mutations conferring rifampicin resistance found in 41 independent Rif-R mutants in the WT and Δ*nucS* strains. The columns show the position of the mutations in the *rpoB* gene sequence, the codon change (modified bases are in bold), the amino acid change caused by each mutation and the number of independent Rif-R mutants isolated from mc^2 155 (WT) or its Δ*nucS* derivative. B) Specificity of base substitutions, summarized by class, produced in the WT and Δ*nucS* strains.

A)

**Position**	**Codon change**	**Amino acid change**	**WT**	**Δ*nucS***
A 1295 G	G**A**C→G**G**C	Asp 432 Gly	0	1
C 1324 T	**C**AC→**T**AC	His 442 Tyr	6	6
C 1324 G	**C**AC→**G**AC	His 442 Asp	2	0
A 1325 G	C**A**C→C**G**C	His 442 Arg	13	20
A 1325 C	C**A**C→C**C**C	His 442 Pro	4	0
G 1334 A	C**G**T →C**A**T	Arg 445 His	0	6
G 1334 C	C**G**T→C**C**T	Arg 445 Pro	1	0
G 1334 T	C**G**T→C**T**T	Arg 445 Leu	2	0
C 1340 T	T**C**G→T**T**G	Ser 447 Leu	9	6
C 1340 G	T**C**G→T**G**G	Ser 447 Trp	2	0
T 1346 C	C**T**G→C**C**G	Leu 449 Pro	0	2
G 1348 A	**G**GC→**A**GC	Gly 450 Ser	1	0
Δ CCAGCTGTC (1275-1283)	CCAGCTGTC	Ser 425 Arg + ΔGlnLeuSer (426-428)	1	0
TOTAL			41	41

B)

**Mutation**	**WT (%) n=41**	**Δ*nucS* (%) n=41**
G:C→A:T	16 (39.0)	18 (43.9)
A:T→G:C	13 (31.7)	23 (56.1)
G:C→T:A	2 (4.9)	0
A:T→T:A	0	0
G:C→C:G	5 (12.2)	0
A:T→C:G	4 (9.7)	0
Deletions	1 (2.4)	0

Supplementary Table 3. Mutation rates of *S. coelicolor* A3(2) M145 and its Δ*nucS* derivatives. Rates of spontaneous mutations conferring rifampicin (Rif-R) and streptomycin resistance (Str-R) of *S. coelicolor* A3(2) M145, its Δ*nucS* derivative and *S. coelicolor* Δ*nucS* complemented with the wild-type *nucS* from *S. coelicolor* ($nucS_{Sco}$). 95% confidence intervals (CI) are indicated. Mut Rate: mutation rate (mutations per cell per generation).

**Strain**	**Genotype**	**Mut Rate Rif-R**	**95% CI**	**Fold**	**Mut Rate Str-R**	**95% CI**	**Fold**
S. coelicolor A3(2) M145	WT	$5.11x10^{-9}$	$2.87\text{-}8.01x10^{-9}$	1	$4.30x10^{-10}$	$0.24\text{-}18.9x10^{-10}$	1
Δ*nucS*	Δ*nucS*	$5.54x10^{-7}$	$4.20\text{-}6.97x10^{-7}$	108.4	$8.49x10^{-8}$	$5.78\text{-}11.4x10^{-8}$	197.4
Δ*nucS*/$nucS_{Sco}$	Complemented	$2.01x10^{-9}$	$0.83\text{-}3.88x10^{-9}$	0.5	$4.30x10^{-10}$	$0.24\text{-}18.9x10^{-10}$	1

Supplementary Table 4. Characteristics of the *M. tuberculosis* representative strains containing NucS polymorphisms. Genome ID/common name, NucS polymorphism, resistance profile, lineage and origin of the strains are shown. Resistance profile indicates not detected/unknown antibiotic resistances (Susceptible) or MDR, multidrug-resistant strain (expressing at least rifampicin and isoniazid resistance).

**GenomeID/ name**	**Polymorphism**	**Resistance profile**	**Lineage**	**Origin**
CDC1551	WT	Susceptible	4	North America
TKK_02_0079	S39R	MDR	4	South Africa
MTB_N1057	S54I	Susceptible	4	South Asia
KT-0040	A67S	Susceptible	2	S. Korea (Broad Inst)
ERR036236	V69A	Susceptible	1	Unknown
BTB 04-388	A135S	MDR	3	Sweden (Broad Inst)
BTB 07-246	R144S	MDR	4	Sweden (Broad Inst)
TKK_03_0044	D162H	Susceptible	4	South Africa
HN2738	T168A	Unknown	Unknown	Unknown (Broad Inst)
MTB_X632	K184E	MDR	4	Central America

Supplementary Table 5. Effect of *M. tuberculosis* NucS naturally occurring polymorphisms on mutation rates. Rates of spontaneous mutations conferring rifampicin resistance (Mut rate, mutations per cell per generation) of *M. smegmatis* Δ*nucS* complemented with the wild-type *nucS* from *M. tuberculosis* (*nucS*$_{TB}$) or the 9 polymorphic alleles. 95% confidence intervals (CI) are shown. Fold change indicates the increase in mutation rate with respect to the strain *M. smegmatis* Δ*nucS* complemented with wild-type *nucS*$_{TB}$ (Δ*nucS/nucS*$_{TB}$), set to 1.

Amino acid change	Codon change	Mut rate	95% CI	Fold change
M. smegmatis Δ*nucS/nucS*$_{TB}$	*nucS*$_{TB}$ from CDC1551	4.17×10^{-9}	$3.29\text{-}5.12 \times 10^{-9}$	1
S39R	AGC→AG<u>G</u>	3.49×10^{-7}	$2.93\text{-}4.0 \times 10^{-7}$	83.7
S54I	AGT→A<u>T</u>T	7.94×10^{-9}	$5.49\text{-}11.07 \times 10^{-9}$	1.9
A67S	GCG→<u>T</u>CG	2.78×10^{-9}	$1.87\text{-}3.84 \times 10^{-9}$	0.7
V69A	GTG→G<u>C</u>G	8.77×10^{-9}	$6.50\text{-}11.2 \times 10^{-9}$	2.1
A135S	GCG→<u>T</u>CG	3.57×10^{-8}	$2.77\text{-}4.38 \times 10^{-8}$	8.6
R144S	CGC→<u>A</u>GC	3.16×10^{-8}	$2.40\text{-}4.0 \times 10^{-8}$	7.6
D162H	GAC→<u>C</u>AC	8.98×10^{-9}	$6.42\text{-}11.80 \times 10^{-9}$	2.2
T168A	ACC→<u>G</u>CC	3.96×10^{-8}	$3.09\text{-}4.81 \times 10^{-8}$	9.5
K184E	AAG→<u>G</u>AG	3.06×10^{-8}	$2.37\text{-}3.75 \times 10^{-8}$	7.3

Supplementary Table 6. Sequence of oligonucleotides used in this work.

Oligonucleotide	Sequence (5'-3')	Purpose
SecMycomar	CCCGAAAAGTGCCACCTAAATTGTAAGCG	Localization of T*n* insertion site
DelnucSm5F	CCCG**CTGCAG**CTGGCCGAGTTCGG	*nucS*$_{Sm}$ *M. smegmatis* deletion
DelnucSm5R	CGGT**AAGCTT**GGCTATCACGAGGCGCACCC	*nucS*$_{Sm}$ *M. smegmatis* deletion
DelnucSm3F	CGGA**AAGCTT**AGCGACGAGTACCGGCTCTT	*nucS*$_{Sm}$ *M. smegmatis* deletion
DelnucSm3R	AATC**GTCGAC**CGAACCCATCAACTTACCGA	*nucS*$_{Sm}$ *M. smegmatis* deletion
CompnucTBF	ACTG**GAATTC**TCGAGTGGTGGCCTTCTCGGATGGCAT	*nucS*$_{TB}$ for Δ*nucS*$_{Sm}$ complementation
CompnucTBR	ACTG**AAGCTT**TCAGAACAGCCGGTACTCGCCGCT	*nucS*$_{TB}$ for Δ*nucS*$_{Sm}$ complementation
CompnucSmF	ACTG**GAATTC**CCGCGCCAGCGAATTGTCGGCGTTCAT	*nucS*$_{Sm}$ for Δ*nucS*$_{Sm}$ complementation
CompnucSmR	CGGC**AAGCTT**TCAGAAGAGCCGGTACTCGTCGCT	*nucS*$_{Sm}$ for Δ*nucS*$_{Sm}$ complementation
RifRRDRrpoBF	GTGGCGGCGATCAAGGAGTTCTTC	RRDR-*rpoB*$_{Sm}$ amplification
RifRRDRrpoBR	GGCGACCGACACCATCTGGCGCGG	RRDR-*rpoB*$_{Sm}$ amplification
DelnucSco5F	GTGT**AAGCTT**CGGCACCGCGGTGAGTGTGC	*nucS*$_{Sco}$ *S. coelicolor* deletion
DelnucSco5R	CGAC**GGATCC**GCGGGCAATGACGAGACGCA	*nucS*$_{Sco}$ *S. coelicolor* deletion
DelnucSco3F	GCTG**GGATCC**TTCTGAGGGCGCGACGCGTC	*nucS*$_{Sco}$ *S. coelicolor* deletion
DelnucSco3R	CAGG**GATATC**TGCCCGCCCTGGTCGGCGAG	*nucS*$_{Sco}$ *S. coelicolor* deletion
CompnucScoF	TACG**GAATTC**GGGTTCTCCTCTCGCACCCCCGACCAGCAGGGG	*nucS*$_{Sco}$ *for* Δ*nucS*$_{Sco}$ complementation
CompnucScoR	TACG**GGATCC**TCAGAACAGCCGCAGCTTGTCGTCCTCGATGCC	*nucS*$_{Sco}$ *for* Δ*nucS*$_{Sco}$ complementation
Rechyg3F	CAGTTTCATTTGATGCTCGATGAG	Recombination. pRhomyco 100%
Rechyg3R	GACTA**ACTAGT**CAGGCGCCGGGGGCGGTGT	Recombination. pRhomyco 100%
Rechyg5F	GATG**CAGCTG**GGAGTGCGCTGTGACACAAGAATCC	Recombination. pRhomyco 100%
Rechyg5R	CGAT**AAGCTT**CGAATTCTGCAGCTCG	Recombi. pRhomyco 100%, 95%, 90% and 85%
Rechyg5intR	GATG**TGATCA**CCGGGTCGGGCTCG	Recombi. pRhomyco 95%, 90% and 85%
Rec95-BclIF	GATG**TGATCA**AGCTGTTCGGGGAGCACTG	Recombination. pRhomyco 95%
Rec95-NheIR	GATG**GCTAGC**GAAGTCGACGATCCCGGTGA	Recombination. pRhomyco 95%
Rec90-85-BclIF	GATG**TGATCA**AGCTCTTCGGGGAACACTG	Recombination. pRhomyco 90% and 85%
Rec90-85-PciIR	GATG**ACATGT**GAAGTCGACGATCCCGGTGA	Recombination. pRhomyco 90% and 85%
S39RF	GGCCGACGGATCGGTCAG**G**GTACATGCTGACGACCG	Site-directed mutagenesis *nucS*$_{TB}$
S39RR	CGGTCGTCAGCATGTAC**C**CTGACCGATCCGTCGGCC	Site-directed mutagenesis *nucS*$_{TB}$
S54IF	CCGTTGAACTGGATGA**T**TCCGCCGTGCTGGTTG	Site-directed mutagenesis *nucS*$_{TB}$

S54IR	CAACCAGCACGGCGGA**A**TCATCCAGTTCAACGG	Site-directed mutagenesis *nucS*$_{TB}$
A67SF	AGAGTCCGGCGGCCAG**T**CGCCAGTGTGGGTGGTCG	Site-directed mutagenesis *nucS*$_{TB}$
A67SR	CGACCACCCACACTGGCG**A**CTGGCCGCCGGACTCTTC	Site-directed mutagenesis *nucS*$_{TB}$
V69AF	CCGGCGGCCAGGCGCCAG**C**GTGGGTGGTCGAGAAC	Site-directed mutagenesis *nucS*$_{TB}$
V69AR	GTTCTCGACCACCCAC**G**CTGGCGCCTGGCCGCCGG	Site-directed mutagenesis *nucS*$_{TB}$
A135SF	GCCGCGAGTACATGACC**T**CGATCGGACCCGTCGAC	Site-directed mutagenesis *nucS*$_{TB}$
A135SR	GTCGACGGGTCCGATCG**A**GGTCATGTACTCGCGGC	Site-directed mutagenesis *nucS*$_{TB}$
A162HF	GCGGCGTGGCGAGATC**C**ACGGCGTGGAGCAGCTGAC	Site-directed mutagenesis *nucS*$_{TB}$
A162HR	GTCAGCTGCTCCACGCCGT**G**GATCTCGCCACGCCGC	Site-directed mutagenesis *nucS*$_{TB}$
T168AF	GCGTGGAGCAGCTG**G**CCCGCTACCTCGAGTTGC	Site-directed mutagenesis *nucS*$_{TB}$
T168AR	GCAACTCGAGGTAGCGGG**C**CAGCTGCTCCACGC	Site-directed mutagenesis *nucS*$_{TB}$
K184EF	GTGCTCGCGCCGGTC**G**AGGGGGTGTTTGCCG	Site-directed mutagenesis *nucS*$_{TB}$
K184ER	CGGCAAACACCCCCT**C**GACCGGCGCGAGCAC	Site-directed mutagenesis *nucS*$_{TB}$
pvv16-seq-fwd	CGGTGAGTCGTAGGTCGGGACGG	PCR amplification and sequencing
pvv16-seq-rev	TGCCTGGCAGTCGATCGTACGCTAG	PCR amplification and sequencing

Supplementary Methods.

Generation of a Δ*nucS* knockout mutant in *M. smegmatis*.

To generate a Δ*nucS* knockout mutant in *M. smegmatis*, a 1.0 kb PstI-HindIII upstream and HindIII-SalI downstream fragments of *nucS* were amplified by PCR, using delnucSm 5F, 5R, 3F and 3R primers, and cloned in-frame into the p2NIL vector. Then, a pGOAL19 PacI-cassette, carrying a β-galactosidase *lacZ* gene, a hygromycin-resistance *hyg* gene and a *sacB* gene, that confers sucrose sensitivity, was also inserted into the p2NIL vector. The resulting plasmid p2NIL-Δ$nucS_{\mathrm{Sm}}$, harbouring an in-frame deletion of the target gene (lacking ≈ 95% of the gene sequence), was electroporated into *M. smegmatis* mc^2 155. Cells were plated on Middlebrook 7H10 agar-X-gal (100 μg ml^{-1}) plus kanamycin (25 μg ml^{-1}) and hygromycin (50 μg ml^{-1}) and incubated for 4-5 days at 37ºC. Single-crossover merodiploid clones were grown in 7H9 broth without antibiotics to allow a second crossover event. Cultures were diluted and counter-selected on Middlebrook 7H10-X-gal (100 μg ml^{-1}) plates containing 2% sucrose and incubated 4-5 days at 37ºC. Double-crossover clones were tested for kanamycin and hygromycin susceptibility to confirm the loss of the plasmid. Finally, *M. smegmatis* mc^2 155 Δ*nucS* colonies were tested by PCR and sequencing to verify the unmarked *nucS* deletion.

Complementation of the *M. smegmatis* Δ*nucS* mutant.

For complementation with *nucS* from *M. smegmatis* mc^2 155, the *MSMEG_4923* gene ($nucS_{\mathrm{Sm}}$), including its own 46-bp promoter region, was amplified by PCR with primers compnucSmF and compnucSmR primers, digested with EcoRI and HindIII and cloned into the integrative vector pMV361, rendering the complementation vector pMV-$nucS_{\mathrm{Sm}}$. Similarly, for complementation with *nucS* from *M. tuberculosis*, the wild-type full-length *MT_1321* gene from CDC1551 control strain ($nucS_{\mathrm{TB}}$), with its own 61-pb upstream promoter region, was amplified by PCR, using compnucTBF and compnucTBR primers, cloned into the pMV361 vector to generate the complementation vector, pMV-$nucS_{\mathrm{TB}}$. Putative complemented mutants were obtained upon electroporation of the plasmids into *M. smegmatis* mc^2 155 Δ*nucS* and incubation of the plated samples on Middlebrook 7H10 agar plus kanamycin (25 μg ml^{-1}) for 3-5 days at 37ºC. Finally, *M. smegmatis* mc^2 155 Δ*nucS* complemented mutants were analysed by PCR and sequencing to verify the proper insertion of the genes.

Generation of Δ*nucS S. coelicolor* knockout mutant and complementation.

pIJ6650 vector was used to clone in-frame two 1.5 kb DNA fragments, one HindIII-BamHI *nucS* upstream fragment plus one BamHI-EcoRV *nucS* downstream fragment, previously amplified by PCR (primers delnucSco 5F, 5R, 3F and 3R). *E. coli* ET12567 (pUZ8002) was transformed with pIJ-Δ$nucS_{Sco}$ (containing the in-frame deletion of the $nucS_{Sco}$ gene) and conjugated with *S. coelicolor* A3(2) M145. Following the isolation of apramycin resistant single-crossover, putative double-crossover mutants were isolated and the unmarked deletion of the $nucS_{Sco}$ gene was verified by PCR. *S. coelicolor* Δ*nucS* was complemented with a wild-type copy of $nucS_{Sco}$ cloned in pSET152. pSET152 is a non-replicative plasmid in *Streptomyces* that carries the *attP* site and the integrase gene of the C31 phage and consequently can integrate into the *attB* site [2] in the chromosome of *Streptomyces*. $nucS_{Sco}$ gene with its own promotor was amplified by PCR with primers compnucScoF and compnucScoR, cloned into pSET152 using EcoRI and BamHI sites to give the pSET-$nucS_{Sco}$ plasmid that was introduced into *E. coli* ET12567 (pUZ8002) by transformation. Finally, pSET-$nucS_{Sco}$ from *E. coli* ET12567 (pUZ8002, pSET-$nucS_{Sco}$) was introduced into *S. coelicolor* Δ*nucS* by conjugation in order to integrate the construction into the chromosome.

Construction of pRhomyco plasmids.

To create a template for measuring homologous and homeologous recombination in *M. smegmatis*, we designed a recombination assay based on the hygromycin-resistance (Hyg-R) gene *hyg*, using the integrative plasmid pMV361. For pRhomyco 100%, a fragment denominated *hyg* 3' (from nucleotide 195 of the coding sequence to the stop codon) of the *hyg* gene, was PCR amplified from pRAM vector, using rechyg3F and rechyg3R primers and digested with EcoRV/SpeI to be cloned in StuI/SpeI targets of pMV361. Additionally, a fragment denominated *hyg* 5′ containing 711 bp (from the start codon of the hyg gene to nucleotide 711) was PCR amplified and cloned using PvuII/HindIII with rechyg5F and rechyg5R primers. Both fragments share two overlapping hyg fragments of 517 bp (nucleotide 195 to 711). The homeologous recombination vectors, named pRhomyco 95%, 90% and 85%, were constructed by replacing the overlapping 517-bp fragment of *hyg* 5′ for synthetic fragments that have 95%, 90% or 85% sequence similarity to the original *hyg* fragment (Supplementary Fig. 2). First, a common fragment to the three of them (from nucleotide 1 to 193 of hyg) was PCR-amplified with rechyg5F and rechyg5intR primers and cloned using PvuII/BclI. Then, each synthetic fragment was PCR amplified and cloned using BclI/NheI with rec95-BclIF and rec95-NheIR primers, in the case of pRhomyco 95%. For pRhomyco 90% and

85%, the variable fragment was cloned using BclI/PcI-BspHI with rec90-85-BclIF and rec90-85-PciIR primers.

Computational analyses.

A summary of all the computational approaches conducted is depicted in Supplementary Fig. 4. For NucS, we used the structure-based alignment of bacterial and archaeal NucS (Supplementary Fig. 3). Then we followed the procedure depicted in Supplementary Fig. 4 to conduct domain analyses using sequences.

Using the different defined regions by structural bioinformatics, we first conducted sequence searches against the large database. We made non-redundant alignments of the N-terminal region (containing 63 sequences) and the C-terminal region (containing 39 sequences) and built profile hidden Markov models (HMMs). These profiles were searched using pHMMER [3] against the large References Proteomes file where additional proteins containing CT and NT regions in alternative proteins were found. While the NT region was found in alternative archaeal proteins (we selected two after checking the alignment F7PKA0_9EURY and L0I7R5_HALRX (DUF91, PF01939), the CT region was found not only in additional prokaryotic groups, but also in eukaryotic sequences (I1F1Q3_AMPQE, I1F1Q5_AMPQE, B3SFT8_TRIAD, B9T981_RICCO, and A0A015J6U4_9GLOM). With the exception of A0A015J6U4, and B9T981, the matching regions are located within a DUF1016 domain (uncharacterized). We checked the alignments to confirm that the catalytic residues, as described in the structure of *P. abyssi* [1], were conserved.

We next searched the PFAM database with NUCS_MYCTU and found a domain, PF01939, which was trained in three NucS full sequences. From the PF01939 domain (539 sequences in 464 species), only 368 entries in 363 (archaeal and bacterial) species gave a match with the full protein (Supplementary data file 1).

To identify MutS and MutL in bacterial and archaeal species, we generated HMMs from different proteins, which would capture most of the bacterial and archaeal diversity (Supplementary Fig. 4). The MutS profile was trained with the following sequences (MUTS_ECOLI, C1F256_ACIC5, MUTS_AQUAE, MUTS_BACTN, MUTS_CHLPN, MUTS_CHLTE, MUTS_CHLAA, WP_027388865.1, MUTS_PROM3, B5YE41_DICT6, MUTS_BACSU, MUTS_STAAM, MUTS_CLOTE, MUTS_FUSNN, C1AEM6_GEMAT, A6DUF8_9BACT, MUTS_RHOBA, MUTS_BRUME, MUTS_NEIMA, MUTS_SALTY, MUTS_VIBC3, MUTS_PSEAE, MUTS_DESVH, MUTS_TREPA, A0A075WU36_9BACT, MUTS_THEMA, WP_009958798.1), while the MutL profile was trained with the following sequences (MUTL_ACIC5, MUTL_AQUAE, MUTL_BACTN, MUTL_CHLPN, MUTL_CHLTE, A9WJ86_CHLAA,

MUTL_DEIRA, MUTL_DICT6, D9S9H6_FIBSS, MUTL_BACSU, MUTL_STAAM, MUTL_CLOTE, Q8RG56_FUSNN, MUTL_GEMAT, A6DHB3_9BACT, Q7UMZ3_RHOBA, MUTL_BRUME, MUTL_NEIMA, A0A0C5TQ33_SALTM, MUTL_VIBCH, MUTL_PSEAE, Q72ET5_DESVH, MUTL_TREPA, A0A075WSF3_9BACT, MUTL_THEMA, and MUTL_ECOLI). The models were searched against each reference proteome where bit scores <50 were discarded to filter and analyze the results. Only full proteins were retrieved by forcing a length > 75%, so partial hits would be excluded.

For actinobacterial and archaeal species not showing NucS, searches in all the particular genomes (only for complete genomes) were done using translated searches against their genomes using tblastn with NucS_MYCTU, MutS_ECOLI, and MUTL_ECOLI for actinobacterial, and NucS_PYRAB, MutS_HALMA, and MUTL_HALSA for archaeal proteins.

References for Supplementary Information

1 Ren, B. *et al.* Structure and function of a novel endonuclease acting on branched DNA substrates. *The EMBO journal* **28**, 2479-2489, doi:emboj2009192 [pii] 10.1038/emboj.2009.192 (2009).

2 Bierman, M. *et al.* Plasmid cloning vectors for the conjugal transfer of DNA from Escherichia coli to Streptomyces spp. *Gene* **116**, 43-49 (1992).

3 Eddy, S. R. A new generation of homology search tools based on probabilistic inference. *Genome Inform* **23**, 205-211 (2009).
